# Post-TB health and wellbeing

**DOI:** 10.5588/ijtld.22.0514

**Published:** 2023-04-01

**Authors:** R. Nightingale, F. Carlin, J. Meghji, K. McMullen, D. Evans, M. M. van der Zalm, M. G. Anthony, M. Bittencourt, A. Byrne, K. du Preez, M. Coetzee, C. Feris, P. Goussard, K. Hirasen, J. Bouwer, G. Hoddinott, M. A. Huaman, G. Inglis-Jassiem, O. Ivanova, F. Karmadwala, H. S. Schaaf, I. Schoeman, J. A. Seddon, T. Sineke, R. Solomons, M. Thiart, R. van Toorn, P. I. Fujiwara, K. Romanowski, S. Marais, A. C. Hesseling, J. Johnston, B. Allwood, J. C. Muhwa, K. Mortimer

**Affiliations:** 1 Department of Clinical Sciences, Liverpool School of Tropical Medicine, Liverpool, UK; 2 Department ofRespiratory Medicine, Liverpool University Hospitals NHS foundation Trust, Liverpool, UK; 3 Department of Infectious Diseases, Liverpool University Hospitals NHS foundation Trust, Liverpool, UK; 4 Department of Respiratory Medicine, Cambridge University Hospital NHS Foundation Trust, Cambridge, UK; 5 Division of Neurology, Department of Medicine, Groote Schuur Hospital, University of Cape Town, Cape Town, South Africa; 6 Health Economics and Epidemiology Research Office, Faculty of Health Sciences, University of the Witwatersrand, Johannesburg, South Africa; 7 Desmond Tutu TB Centre, Department of Paediatrics and Child Health, Faculty of Medicine and Health Sciences, Stellenbosch University, Tygerberg, South Africa; 8 University Hospital, University of Sao Paulo School of Medicine, Sao Paulo, SP, Brazil; 9 Department of Thoracic Medicine, St Vincent’s Hospital Clinical School University of New South Wales, Sydney, NSW, Australia; 10 Division of Physiotherapy, Department of Health and Rehabilitation Sciences, Faculty of Medicine and Health Sciences, Stellenbosch University, Tygerberg, South Africa; 11 Occupational Therapy Department, Windhoek Central Hospital, Ministry of Health and Social Services, Windhoek, Namibia; 12 Division of Occupational Therapy, Department of Health and Rehabilitation Sciences, Stellenbosch University, Tygerberg, South Africa; 13 Faculty of Medicine and Health Sciences, Stellenbosch University, Tygerberg, South Africa; 14 Paediatric Pulmonology, Department of Paediatrics and Child Health, Faculty of Medicine and Health Sciences, Stellenbosch University, Tygerberg, South Africa; 15 Department of Psychiatry, University of the Witwatersrand, Johannesburg, South Africa; 16 University of Cincinnati College of Medicine, Cincinnati, OH, USA; 17 Division of Infectious Diseases and Tropical Medicine, Medical Centre of the University of Munich, German Centre for Infection Research, Partner Site Munich, Munich, Germany; 18 TB Proof, Cape Town, South Africa; 19 Department of Infectious Diseases, Imperial College London, London, UK; 20 Department of Paediatrics and Child Health, Faculty of Medicine and Health Sciences, Stellenbosch University, Tygerberg, South Africa; 21 Division of Orthopaedic Surgery, Faculty of Medicine and Health Sciences, Stellenbosch University, Tygerberg, South Africa; 22 Task Force, Global Plan to End TB, 2023–2030, Stop TB Partnership, Geneva, Switzerland; 23 Department of Medicine, University of British Columbia, Vancouver, BC, Canada; 24 Provincial TB Services, BC Centre for Disease Control, Vancouver, BC, Canada; 25 Neurology Research Group, Neuroscience Institute, University of Cape Town, Cape Town, South Africa; 26 Division of Pulmonology, Department of Medicine, Faculty of Medicine, Health Sciences, Stellenbosch University, Cape Town, South Africa; 27 Department of Medicine, Therapeutics, Dermatology and Psychiatry, Kenyatta University, Nairobi, Kenya; 28 Department of Medicine, University of Cambridge, Cambridge, UK; 29 Department of Paediatrics and Child Health, College of Health Sciences, School of Clinical Medicine, University of KwaZulu-Natal, Durban, South Africa

**Keywords:** quality of life, post-tuberculosis lung disease, tuberculous neuropathy, tuberculous pericarditis, post-TB socio-economic burden

## Abstract

TB affects around 10.6 million people each year and there are now around 155 million TB survivors. TB and its treatments can lead to permanently impaired health and wellbeing. In 2019, representatives of TB affected communities attending the ‘1^st^ International Post-Tuberculosis Symposium’ called for the development of clinical guidance on these issues. This clinical statement on post-TB health and wellbeing responds to this call and builds on the work of the symposium, which brought together TB survivors, healthcare professionals and researchers. Our document offers expert opinion and, where possible, evidence-based guidance to aid clinicians in the diagnosis and management of post-TB conditions and research in this field. It covers all aspects of post-TB, including economic, social and psychological wellbeing, post TB lung disease (PTLD), cardiovascular and pericardial disease, neurological disability, effects in adolescents and children, and future research needs.

## Abstract (fr)

La TB touche environ 10 millions de personnes chaque année et il y a aujourd’hui environ 155 millions de survivants de la TB. La TB et ses traitements peuvent entraîner des dommages permanents à la santé et au bien-être. En 2019, les représentants des communautés touchées par la TB participant au « 1^er^ Symposium international post-tuberculose » ont appelé à l’élaboration des directives cliniques sur ces questions. Cette déclaration clinique sur la santé et le bien-être post-TB répond à cet appel et s’appuie sur le travail du symposium, qui a réuni des survivants de la TB, des professionnels de la santé et des chercheurs. Notre document offre une opinion d’expert et, dans la mesure du possible, une orientation fondée sur des données probantes pour aider les cliniciens dans le diagnostic et la prise en charge des pathologies post-TB et la recherche dans ce domaine. Il couvre tous les aspects post-TB, y compris le bien-être économique, social et psychologique, la maladie pulmonaire post-TB (PTLD), la maladie cardiaque et péricardique, la déficience neurologique, les effets chez les adolescents et les enfants, et les besoins futurs de recherche.

In 2020, there were approximately 10 million cases of TB, with 1.5 million recorded deaths.^1^ Thanks to new and continually improving diagnostics and treatments, there are now approximately 155 million TB survivors worldwide.^2^ However, TB and its treatments can leave a person with permanently damaged tissues. Survivors typically experience a transition from acute illness to living with multifaceted chronic disease. Post-TB morbidity affects adults and children and varies widely. It can include physical conditions such as post-TB lung disease (PTLD), neurological impairment, cardiac disorders and psychiatric illness. Many survivors also experience ongoing psychosocial and economic effects, which also need to be addressed as part of a system-wide approach to improving survivors’ quality of life.^3^ The true burden of post-TB conditions is not fully known due to lack of epidemiological data, particularly from low- and middle-income countries (LMICs). However, with PTLD alone estimated to affect 18–87% of survivors, and with a known 10 million people affected with TB each year, TB is a leading cause of chronic disease worldwide.^4^ Furthermore, it is probable that delayed case-finding and treatment of TB due to COVID-19 will lead to more individuals suffering from the consequences of post-TB condition for years to come

## AIMS AND SCOPE

**Figure 1:**
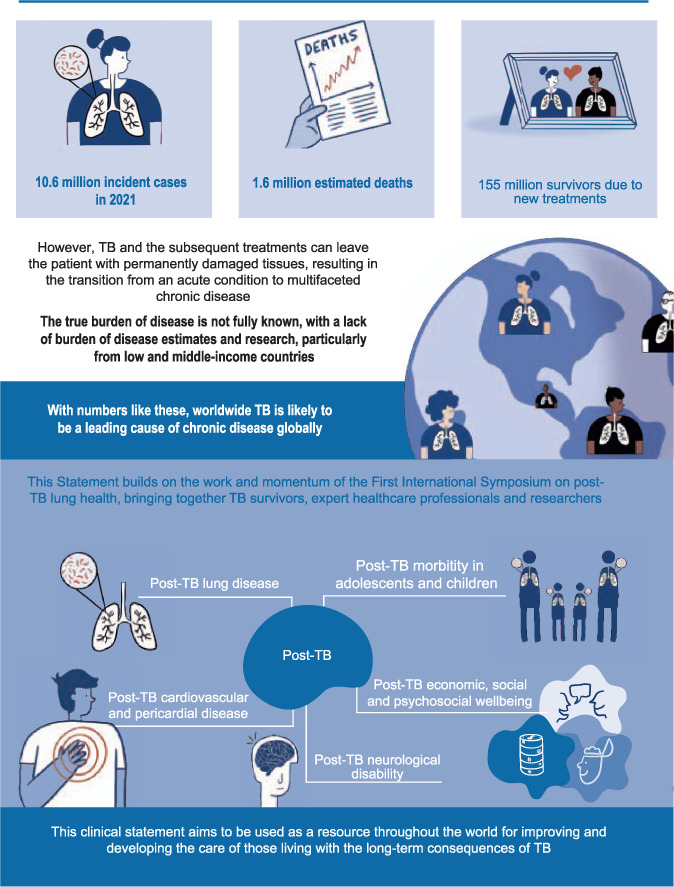
Post-TB: visual abstract of the clinical statement.

This clinical statement builds on the work and momentum of the 1^st^ International Post-Tuberculosis Symposium held in Stellenbosch, South Africa, in 2019.^5^ We aim to provide a preliminary guide to aid clinicians in the diagnosis and management of post-TB conditions and to support research in this important field. This clinical statement covers post-TB economic, social and psychological wellbeing, PTLD, post-TB cardiovascular and pericardial disease, post-TB neurological disability, post-TB effects in adolescents and children, and research needs. Figure 1 summarises the information and guidance in this clinical statement.

## METHODOLOGY

### Multidisciplinary working group

**Figure 2:**
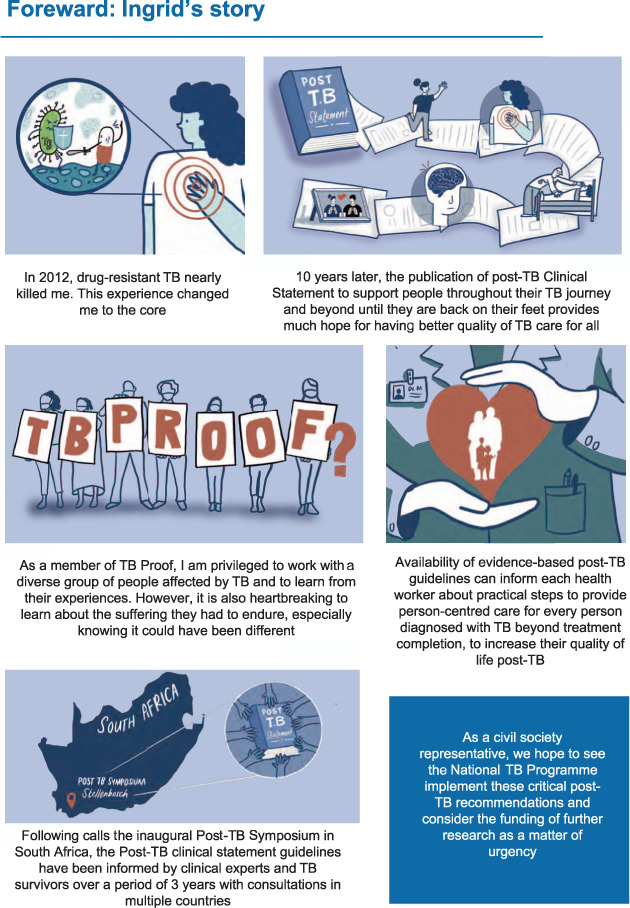
Foreword – a TB survivor’s perspective, Ingrid Schoeman, TB Proof. TB Proof is a TB advocacy group based in South Africa. Ingrid Schoeman is a TB survivor and co-author of this Clinical Statement.

The clinical statement working group was chaired by KM, JM and RN. Working group members, authors and contributors included TB survivors (Figure 2), expert healthcare professionals and researchers. Testimony in the form of anonymised quotes from TB survivors consulted during the process of developing the clinical statement is included with their permission. The statement was developed through roundtable discussions according to the scope agreed at the 1^st^ International Post-Tuberculosis Symposium.^5^

### Literature review on lived experience of post-TB health and wellbeing

**Figure 3:**
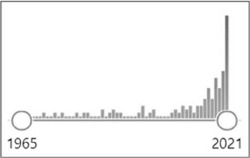
Rate of publications on post-TB health and well-being, 1965–2021.

There is limited evidence on the full spectrum of complications that people experience after microbiological cure for TB. We conducted a rapid review of peer-reviewed post-TB literature to gain an understanding of the available evidence on economic, psychological, and social impacts to inform section 1 (Supplementary Data). We noted a marked increase in the yearly rate of publications on post-TB since 2005 (Figure 3).

### Consensus clinical guidance

At the scoping stage, the working group noted a lack of large prospective observational or interventional studies to guide clinical recommendations on some areas, such as PTLD. The clinical guidance on the assessment and management of PTLD, post-TB cardiovascular and pericardial disease, post-TB neurological disability and post-TB effects in adolescents and children was therefore based on expert opinion, informed by published evidence. We took this approach because of the urgent need to develop interim guidance to meet TB survivors’ needs, which would have been unduly delayed by conducting formal systematic reviews in a field with little high-quality evidence.

### Drafting and review

Each section was drafted by working group members sub-grouped according to their area of expertise. Drafts were reviewed by all authors and chairs before peer review. This clinical statement will be updated as more evidence becomes available. Articles retrieved from PubMed using the search strategy are shown in Supplementary Data 1.

## 1. POST-TB ECONOMIC, SOCIAL AND PSYCHOLOGICAL WELLBEING

The full spectrum of economic, psychological and social complications experienced beyond TB treatment is not fully understood. Up to half of TB survivors have some form of chronic pulmonary dysfunction despite microbiologic cure, impairing their quality of life.^5^–^11^ These post-TB consequences are likely to impose a continued financial burden on TB survivors, their families, their communities and the health system.^5^ The WHO defines health as a state of ‘complete physical, mental and social well-being’.^12^ Accordingly, the long-term psychological, social and economic well-being of TB survivors needs to be considered as essential, and must be addressed in addition to chronic physical impairment. The psychological and social impacts of TB diagnosis and treatment have been well described:^13^–^15^ and people diagnosed with TB report psychological distress, pain, stigma, decreased exercise capacity and poor health-related quality of life (HRQoL) at TB treatment completion.^16^–^18^ However, there is limited evidence on the psychological and social impact of TB illness following microbiological cure.

### Complications experienced beyond TB treatment

#### Health-related quality of life

Despite microbiological cure, TB can continue to negatively affect an individual’s quality of life at the end of TB treatment.^18^–^23^ People with lived experience of TB describe debilitating after-effects:

Unfortunately, many individuals, like myself, suffer for years after treatment completion to battle against side effects of the medication. The toxicity of the medication is overlooked, and more care should be given to post-TB health and well-being of every TB survivor. (Female TB survivor, 24 years)

Nowadays, I cannot walk long distance as I used to do, my legs are weak as well as my whole body is weak. I agree that I am healed because they tested me and said I don’t have TB – but my whole body is still weak. During cold it is worse I feel pain all over my body. (Female TB survivor, 40 years)

The effects are really serious. Once you get TB it impairs your health as well as your ability to carry out your daily activities. (Male TB Survivor, 54 years)

However, as you can see now I am healthy and OK although I am not yet to my normal life like before, but for me I see my progress is good and I have started doing my activities. (Female TB survivor, 35 years)

An individual’s perception of their HRQoL is typically lowest at the start of TB treatment but improves during treatment.^15^,^16^ However, an estimated 20–50% of TB survivors develop lung impairment after completing treatment,^6^,^20^,^24^ potentially leading to progressive deterioration in quality of life.^19^,^21^,^25^ Persistent symptoms, economic losses and impaired social life due to TB are significantly associated with poor post-TB HRQoL.^26^ The long-term adverse effects of anti-TB medication may also reduce a TB survivor’s HRQoL.^27^ Moreover, people with drug-resistant TB (DR-TB) often face more severe consequences due to the long duration of treatment; the treatment-related adverse effects of second-line TB regimens are often described as worse than the disease itself.^13^ Apart from a few reports, we know very little about the full spectrum of economic, social and psychological complications experienced beyond treatment for DR-TB. In one of the few studies reporting long-term follow-up of people diagnosed with multidrug-resistant TB (MDR-TB), participants still reported experiencing poor HRQoL 18 months after cure.^7^ PTLD resulting from long-term physical, psychological, social and economic consequences may contribute to prolonged poor HRQoL experienced after TB treatment,^28^ and may explain why some TB survivors never report HRQoL similar to that of comparator groups without TB.^16^

#### Mental health and psychological impact

Poor mental health is becoming increasingly recognised in individuals with chronic physical comorbidities.^14^ Thornicroft et al. estimated that rates of depression and anxiety are at least two-fold higher in those with TB than in the non-TB-infected population.^29^ Mental health disorders associated with TB disproportionately affect women and people living in LMICs.^30^ The pooled prevalence of depression is higher among people with MDR-TB (52%) than among those with drug-susceptible TB (45%), and significantly correlates with severity and duration of disease.^30^–^33^ The greater effects on mental health are linked, not only with the treatment itself, but with loss of income, impact on social roles and a greater experience of hopelessness and stigma.^34^,^35^ Almost a third of people undergoing treatment for TB report anxiety and depressive symptoms at the start of treatment.^16^,^36^ Moreover, TB treatment itself may cause adverse psychiatric events.^14^ While depression and anxiety generally improve during TB treatment,^37^ untreated depression can reduce treatment adherence and result in poorer TB treatment outcomes, impaired functioning, decreased quality of life and greater disability that may last for years after treatment completion.^36^,^38^ One of the goals of the Stop TB Partnership is to integrate mental health services into TB care.^39^ In a field report from India that assessed the wellbeing of people undergoing TB treatment, only 54% of people enrolled in treatment reported a ’happy mental status’ at the end of treatment.^10^ Therefore, it is vital to intervene early with support for the individual, family and community, such as screening for mental health problems and counselling offered at diagnosis, to improve treatment outcomes. TB and mental health disorders also share common risk factors, including unstable housing, HIV-positive serology and alcohol/substance use disorders.^14^,^40^ The link between alcohol use disorder, TB, and mental health has been well established and highlights the need to systematically screen for early identification of mental health or alcohol use disorders in all people engaged with prevention and treatment services for TB.^40^–^42^ Unless the social determinants of TB are addressed, TB survivors will remain at risk for adverse health outcomes like TB re-infection and a higher risk of death.^43^,^44^

#### Physical functioning, exercise capacity and sleep disturbance

TB sequelae include impaired lung function, chronic respiratory failure, sleep disorder and pulmonary hypertension.^45^ Lung impairment after TB treatment is a common outcome, leading to poor physical functioning and reduced exercise capacity, with a reduction in quality of life.^18^,^46^ Reduced exercise capacity after treatment completion has been described in several international studies.^17^,^18^,^47^–^49^ Impaired lung function not only impacts quality of life but also has economic implications if poor physical functioning affects ability to return to productive employment:^20^,^26^

For now, when I am trying to work, I can hardly work for two hours – not more than that – while in the past I was working twelve hours. . . (Male with TB, 38 years)

Ah, the body does not come back like in the beginning, because in the beginning I used to work without coughing, I did not select type of work, but nowadays I am very carefully by selecting the type of work I am involved myself in, so that I protect my chest. (Male TB survivor, 31 years)

People with TB may also experience insomnia or sleep disturbances at diagnosis or during treatment, often co-occurring with other conditions such as depression, anxiety disorders and post-traumatic stress disorder, which negatively affect HRQoL.^50^,^51^ Sleep disorder has also been identified among the common post-treatment TB sequelae.^45^ Sleep deprivation can worsen TB or place individuals at risk for pulmonary TB (PTB).^52^ Assessing sleep quality and addressing poor sleeping patterns can help improve overall health.

#### Stigma and its consequences

TB continues to be a stigmatised disease.^39^ Stigma has a negative impact on health outcomes, affecting health-seeking behaviour and progression along the TB care pathway.^53^–^56^ TB stigma also has serious socio-economic consequences, particularly for women. It can result in isolation, helplessness, disruptions in social networks, a breakdown in personal relationships, and ongoing work/school interruptions:^45^,^57^

Having TB is one of the most challenging things a person has to go through, one’s life changes completely. I was a first-year university student at the time I got diagnosed, not knowing that it will take 7 years for me to return to university. . . The internal stigma is also an issue, I was questioning myself if I was even capable of going back to university, well having had a ’poor man’s disease’ as it was mostly known as the disease of the ’other’. (Female TB survivor and advocate, 31 years)

Stigma also prevents economic recovery following TB treatment completion.^57^ People with DR-TB often experience more self-stigmatisation (blame and shame) because resistance is often associated with non-adherence.^58^ Treatment side effects can expose these individuals to further stigma due to mental health problems, disability and poverty.^13^ There is limited evidence on the long-term sequelae of TB stigma. Adolescents often report long-lasting internalised stigma after DR-TB treatment.^59^ Rajeswari et al. reported that at the end of treatment, 47% of people still experienced effects of stigma.^10^ Only 54% of men and 52% of women reported feeling happy most of the time, and social stigma persisted beyond completion of treatment.^10^ People-centred support and better communication between health-care professionals and the individuals they are treating may help to reduce the stigma surrounding TB and PTLD.^60^,^61^ Counselling may also benefit adolescents and adults struggling with depression and poor self-esteem due to prolonged suffering and disability.^62^

#### Impact on children

Children represent 11% of the global TB burden, but there are few data available on the long-term consequences of TB in children. Only two articles were identified that assessed impacts in children with TB,^62^,^63^ and neither addressed post-acute effects. The need to understand the impact of TB on long-term respiratory morbidity has been previously highlighted.^5^ However, there is also a need to understand the long-term social consequences of TB in children. Aspects that need to be explored include the long-term impact of TB on school performance, school attendance, child development, physical growth, experience with stigma and discrimination, and the prevalence of depression and anxiety after TB treatment completion. There is also a need to understand the impact of TB on child contacts as child contacts may experience social and economic consequences when their caregivers are diagnosed and receive treatment for TB:

In the middle of treatment my situation changed, I failed to meet the needs of my children, but right now, I am struggling, though not as it was when I was not sick, but I tried my level best to make sure my children get their needs, although sometimes they eat only one meal while we sleep. (Male TB Survivor, 38 years)

#### Contribution of post-treatment disability to TB burden of disease

**Figure 4:**
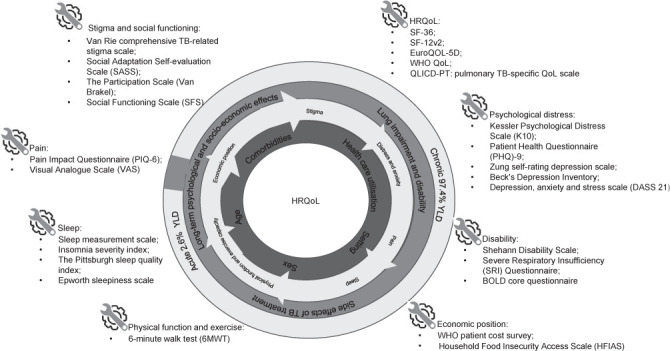
Modelling and measuring post-TB wellbeing: schematic showing some of the psychological and socio-economic consequences that lead to a poor perception of HRQoL and the tools used to measure these. HRQoL = health-related quality of life; SF = Short Form (Health Survey); YLD = years lived with a disability.

Conditions present at treatment completion and those that persist beyond treatment completion contribute to the total burden of TB disease. A measure of the global burden of disease, disability-adjusted life-years (DALYs), is estimated by combining the burden of mortality (i.e., years of life lost [YLL]) and burden of morbidity (i.e., years lived with a disability [YLD]) due to a disease or condition. DALYs can be used to compare the overall health and life expectancy of diseases that cause premature death but little disability to those that do not cause death but disability. In TB, DALYs can be used to estimate how much post-TB mortality and morbidity contribute to the total burden of TB disease. Current estimates for TB assume that individuals return to ‘perfect health’ (i.e., have no disability) at the end of TB treatment, thus underestimating the burden of TB disease and the cost effectiveness of interventions aimed at prevention and early diagnosis.^64^–^66^ The literature identified in our rapid review documents compelling evidence that TB survivors have a higher risk of death and an average of 3.6 more years of potential life loss than the general population or matched controls without active TB despite adequate TB treatment.^44^,^67^–^71^ Quaife et al. reported that the estimated burden of TB increased by an additional 6.1 million DALYs (54% increase) when post-TB mortality and morbidity was considered.^66^ When they considered other TB conditions, this increased to 20.1 million DALYs. Their results highlight the need to consider mortality and disability after treatment completion (post-TB sequelae) to better estimate the total burden of TB disease.^66^ This finding has been echoed by others, confirming that DALYs attributed to PTLD represent about 50% of the total burden estimate.^72^ Other researchers have estimated that YLL contribute approximately one-quarter and YLD contribute three-quarters (77%) of the total burden of disease associated with pulmonary impairment after TB.^64^ Chronic pulmonary impairment after TB accounts for 97.4% of YLD, while illness before completion of treatment, including those from acute treatment-related side effects accounts for the remainder of YLD (Figure 4).^64^ Recent evidence from Blantyre, Malawi, demonstrated that over 90% of projected lifetime YLD was concentrated within the most severely affected 20% of survivors.^65^ Per-case post-TB burden estimates are greater among women, younger people, HIV-positive individuals and those from countries with high-incidence rates.^65^,^72^ A model of some of the psychological and socioeconomic consequences that contribute to poor HRQoL, examples of tools used to measure these and how PTLD contributes to the burden of TB disease is shown in Figure 4.

#### Economic consequences of TB

There is little evidence on how costs related to PTLD impact financial status or to what extent this contributes to the proportion of people with TB and their households facing catastrophic total costs (costs that account for 20% or more of the individual’s annual household income). Contributors to catastrophic total costs include direct medical expenditures, non-medical expenditures and overall indirect costs (i.e., loss of income due to loss of productivity or inability to work).^39^,^73^ People undergoing treatment for TB incur significant costs and dissaving (borrowing money, selling assets or using savings) to cover healthcare costs during TB illness.^20^,^74^ This financial burden is significantly greater for people with DRTB.^75^ People with extrapulmonary TB (EPTB) may continue to experience the financial burden of TB after treatment completion, as they are more likely to be hospitalised post-diagnosis and have a longer mean duration of treatment.^76^ Costs due to TB have severe catastrophic economic repercussions, pushing individuals and their households into extreme poverty:^74^–^78^

After getting into this situation, life has become difficult because of this disease. I am unable to work productively. I always use money I had but there is no way to return those money I used. (Male with TB, 48 years)

Most current estimates of the economic burden of TB are based on the assumption that after treatment no further costs are incurred and that productivity is not diminished.^66^ However, preliminary findings of a longitudinal cohort study in three sub-Saharan African countries suggest that while employment, hours worked and savings improve on treatment, further recovery during the 6 months after treatment completion is limited.^79^ TB had a ‘serious’ or ‘very serious’ impact on participants’ financial status 6 months after completing treatment. A study conducted in Malawi found that TB-affected households remain economically vulnerable even at 12 months after TB treatment completion, with more individuals living in poverty (earning <US$1.90/day) 12 months after TB treatment completion, compared with their income before their TB diagnosis.^20^,^80^ Chest symptoms interfering with work and causing ongoing school interruptions were also noted in the year after TB treatment completion.^20^ At 3-year follow-up, 20% of participants still experienced symptoms and 28% showed abnormal lung function on spirometry.^81^ The emergence of mental illness during or post-TB treatment further contributes both directly and indirectly to income loss through increased health expenditure and reduced productivity.^82^ The indirect costs of TB due to effects on mental health far exceed the direct costs, strengthening motivation for mental health screening both before and after TB treatment. Economic recovery after completing TB treatment could be promoted through strategies like counselling and guidance to improve employment placement, training, job placement, or services to support job retention for individuals, particularly those with disabilities.^35^,^39^ TB survivors describe loss of employment as a major problem:

Patients are forced to leave employment as soon as they start showing symptoms for TB and have severe difficulty in getting back into work after treatment has been completed. As someone who was employed before symptoms for TB began, the rapid rate at which I lost my job as soon as I began to show symptoms of TB was outrageous... Unfortunately, during TB treatment, it is naturally very difficult to get into a work routine, but more so post-TB treatment. It becomes extremely difficult to get employed again after such a huge gap in work. In addition, side effects from the TB treatment can often last for years after treatment has been completed, which results in individuals being unable to be re-employed... not enough is being done to support TB patients, especially financially, once treatment is completed. (Female TB survivor, 24 years)

In the beginning, I used to do my small business to earn me a living, but now I do nothing. (Male TB survivor)

Sweeney et al. noted that, while the financial burden of PTLD is probably lower than the burden before or during treatment, it is still significant for people and should not be ignored.^83^ The financial burden experienced after treatment completion should be considered when estimating the proportion of households experiencing total TB-related costs. This additional burden could further contribute to downward economic drift, leading to increased risk for TB reinfection and mental illness, and promoting a vicious cycle of ill-health and poverty.^83^ For young people, the financial costs of TB and recovery may disrupt their studies and delay their careers:

As someone who was diagnosed with multidrug-resistant TB at the beginning of my second year in my undergraduate degree, being isolated in hospital for 50 days meant that I was forced to take a break from my education for a while. . .. For many students, it is not easy to go back into education; some may be forced to start working, as there is a shortage of financial help for them to survive. (Female TB survivor, 24 years)

### Longitudinal research: TB sequel study in four African countries

TB Sequel (NCT03251196) is an example of a multi-country, multi-centre, observational cohort study designed to understand the pathogenesis and risk factors for PTLD in South Africa, Mozambique, Tanzania and The Gambia.^84^ Adults (≥18 years) with PTB were recruited at initiation of TB treatment between September 2017 and December 2019 and followed prospectively for a minimum of 24 months. Participants were treated according to the local standard of care guided by their national TB programmes. The following were collected from all participants at baseline (0M) and defined study visits: clinical data, data on risk factors, comorbidities and socio-economic status, and sputum, urine and blood samples.^84^ At 0, 2, 6, 12 and 24 months, trained study staff administered the validated HRQoL measurement tool (Short-Form 36 Health Survey [SF-36]), the pain impact questionnaire (PIQ-6TM) for pain, the Kessler Psychological Distress Scale (K10) for psychological distress, the Van Rie et al. (2008) Comprehensive TB Stigma Assessment Scale,^85^ and an adapted version of WHO’s TB Patient Cost Survey Tool.^86^ Preliminary analysis of data from the subgroup with drug-susceptible TB demonstrated significant effects on mental health, physical health and economic wellbeing:

#### Effects on mental health

Although HRQoL and psychological distress improved during TB treatment, approximately 15% of people continued to report psychological distress and a low mental component summary score and after treatment completion,^87^ with mental health rated below the population average.^88^

#### Social life

At treatment completion, 54% of the group reported that TB continued to disrupt their participation in work or school, 18% reported that TB continued to cause disruptions to family life and 9% reported continued disruption to social life.^89^,^90^

#### Physical health

Some individuals develop pulmonary impairment after completing TB treatment (see Section 2: Post-TB lung disease), affecting their HRQoL.^19^ Consistent with other studies, TB Sequel reported a relationship between a lower HRQoL disease severity measured by the Falk or Ralph score on chest X-ray (CXR) at treatment completion.^18^,^91^–^93^ Those with a Falk score indicating moderate or far advanced disease were 1.5 times more likely to have a low physical component summary score at treatment completion than those with a score indicating no or minimal TB disease.^91^

#### Economic implications

Analyses to explore recovery in employment and saving after treatment completion showed that employment and hours worked per week were low at the start but improved with TB treatment, with the biggest change observed at treatment completion and limited further recovery 6 months later. At 6 months after treatment completion, less than 10% of participants reported experiencing dissaving, but those who did reported heavy losses.^79^ Individuals who perceived the financial impact of TB to be ‘moderate’, ‘serious’ or ‘very serious’ at treatment completion were less likely to return for their scheduled follow-up study visit 6 months after treatment completion.

### The need for comprehensive post-TB recovery programmes

TB survivors have advocated for comprehensive care beyond treatment completion to improve their quality of life. Care should be provided for up to 2 years post-treatment completion, with an evaluation every 6 months, including a mental health assessment.^5^,^94^

#### Integration of post-TB services with other healthcare

Complete integration of HIV and post-TB services for individuals may be a viable strategy to manage the high number of TB survivors who face ongoing disability and elevated mortality risks.^72^ The TB treatment period may also be an opportunity to promote smoking cessation^95^ and screening for common non-communicable diseases, undernutrition, alcohol misuse disorder and mental health disorders.^96^ This screening could be incorporated into comprehensive care packages that address the cardiovascular, socio-economic and psychological comorbidities faced by TB survivors.^97^ Pragmatic screening for HIV, hypertension, diabetes mellitus (DM) and renal disease is also feasible and offers the chance to link those identified to appropriate pathways and ongoing care.^98^,^99^ A simple mental health screening questionnaire also appears to be an effective and scalable intervention that can be implemented within TB programmes.^100^ The Patient Health Questionnaire (PHQ-9), designed to detect common mental disorders, has been widely studied and recommended for use in primary health care.^101^ The information–motivation–behavioural skills model^102^ has been proposed as the basis for a strategy to address HIV-related risk behaviour within long-term TB care, while addressing additional factors impacting adherence, such as health beliefs, education and economic barriers.^34^

#### Pulmonary rehabilitation

Lung function tests have been recommended to detect and specify possible lung function impairments in TB survivors.^5^ Preliminary data from studies in both high- and low-income settings suggest that pulmonary rehabilitation programmes for people with PTLD are viable and associated with improved quality of life, exercise capacity and respiratory outcomes.^103^,^104^ In addition to psychosocial benefits, pulmonary rehabilitation reduces hospital days and the utilisation of costly healthcare resources due to improved lung function and self-management.^105^

#### Financial and psychosocial support

Social protection programmes that provide food and financial support may help mitigate the long-term consequences of TB and reduce the direct and indirect costs of treatment.^106^ Further research is needed to help understand if these programmes help TB survivors avoid long-term catastrophic costs:

There are multiple ways in which an individual who is suffering with life after TB, can be supported. Interventions such as patient support packages can lead to patients feeling like they are supported after treatment...check up on their health and mental well-being. Financial aid would be a huge and beneficial support to TB survivors in order to get them back on track whilst they are searching for job opportunities. (Female TB survivor, 24 years)

There is also an opportunity to involve TB survivors in designing and delivering support programs. These ‘‘TB champions’’ can create awareness of post-TB consequences and support others in their community, by sharing information and their experiences while advocating for high-quality post-TB care:^107^

I lost nine members of my family to TB-HIV. . .. Neither the children nor adults are TB nor HIV advocates in the family. I am the only one in a family of 32 fighting TB and HIV. . . (Male TB survivor)

Psychosocial support has been identified as one of the most important factors affecting quality of life among individuals with DR-TB.^108^

#### Towards people-centred outcome measures

Microbiological cure, treatment completion, loss to follow-up and death during treatment are routinely measured by national TB programmes, but people-centred outcomes that reflect the health and well-being of TB survivors, their families and community are lacking.^109^ Incorporating long-term indicators as part of routine care would help ensure that the long-term health of TB survivors should be considered as an integral part of TB care. Evaluations should include measures of HRQoL, socio-economic consequences, co-exposures and comorbidities.^5^ With guidance, standardised measurement of post-TB economic, social and psychological wellbeing can be conducted within national TB programmes.^5^ However, reporting mechanisms will need to be adapted to capture the full spectrum of complications and the impact of interventions implemented to improve post-TB outcomes.

### Key messages

The literature on post-TB experiences highlights four key messages:

- There is limited evidence on the psychological and social impact of post-TB morbidity. More research is needed to enable better care of survivors;
- PTLD contributes to the total burden of disease and should not be ignored;
- There is limited evidence on the economic consequences and catastrophic costs associated with PTLD;
- Programmatic interventions are needed to optimise long-term health among TB survivors. To address the areas of need identified in our rapid literature review, TB guidelines should go beyond TB treatment. Improving the HRQoL and socioeconomic recovery of TB survivors should be considered important secondary goals of national TB programmes. PTLD contributes to the total burden of TB disease and should therefore not be overlooked.

## 2. POST-TB LUNG DISEASE

PTLD encompasses lung disease(s) and pathologies occurring after one or more episodes of PTB.^5^ It is defined as ‘evidence of a chronic respiratory abnormality, with or without symptoms, attributable at least in part to previous TB’.^5^ Single or recurrent episodes of PTB can affect an individual’s lung health and may cause disabling symptoms that significantly impact their long-term health. Chronic TB sequelae can adversely affect a TB survivor’s quality of life, with negative impacts on psychological, social and economic outcomes, and reduce overall life expectancy.^30^,^71^,^110^,^111^ PTLD is recognised as one of Africa’s ‘big five’ respiratory diseases,^112^ affecting at least half of TB survivors in some studies.^11^ The type of lung damage that has been documented among people experiencing TB is complex and highly variable.^110^,^113^,^114^ Structural abnormalities include bronchiectasis, bronchial stenosis, cavitation, fibro-nodular scarring and pleural thickening. This damage results in alterations in lung compliance, gas exchange, functional lung volumes and airflow. Consequent lung function abnormalities include restrictive, obstructive and mixed defects, with the risk of irreversible respiratory impairment increasing with each episode of PTB.^110^ With an estimated 155 million TB survivors alive in 2020,^115^ the potential disease burden of post-TB-specific morbidity is very large. Among people who were diagnosed with TB during 2019 alone, PTLD accounted for an estimated 58 million DALYs.^72^ This indicates the urgent need for PTLD to be recognised as a leading cause of chronic lung disease, and for more research into its diagnosis, pathophysiology and optimal person-centred management to reduce morbidity and achieve better TB outcomes for survivors.^68^,^111^,^116^ The guidance on PTLD in this practice statement builds on the Global Plan to End TB^39^ and on the clinical standards for PTLD^46^ recently published by the International Union Against Tuberculosis and Lung Disease (The Union), which represented the first formal attempt to develop a consensus-based approach to this significant global challenge.

### Clinical presentation of PTLD

#### Severity and symptom pattern

The severity and clinical presentation of PTLD is highly variable. Individuals may suffer chronic daily symptoms, be oligo- or asymptomatic, or symptoms may present only when there is exacerbating inter-current illness. Health workers providing care for TB survivors need to be able to assess and manage the broad spectrum of PTLD. Pathology may reflect shared risk factors for TB and other chronic lung disease, including chronic obstructive pulmonary disease (COPD), such as smoke exposure and childhood respiratory infections. Some people may have had chronic lung disease (diagnosed or undiagnosed) before the diagnosis of TB. TB is also an independent risk factor for TB-associated obstructive pulmonary disease (a form of COPD),^117^ bronchiectasis and other manifestations of chronic lung disease. The risk of exacerbations and hospitalisations among people with PTLD is unclear, due to a lack of high-quality prospective studies. However, these risks are likely to be increased, based on extrapolation of evidence from studies in people with smoking-related COPD.^118^ PTLD and its consequences can be broadly divided into four main severity categories (Table 1).

**Table 1:** Proposed severity classification of PTLD^*^

Category	Description	Prognosis^*^
Not detected	Does not meet the definition of PTLD	Effects on future lung health, symptoms and survival not well defined
	No detectable abnormality on lung function testing or chest imaging	Normal future lung health and survival can be expected
Mild	No or minimal symptoms	Possibility of accelerated decline in lung function and increased risk of future lung pathology and exacerbations
	Normal lung function	
	Normal or minimal structural lung disease detected on chest imaging	
Moderate	Variable symptoms	Increased risk of accelerated decline in lung function, future lung pathology, exacerbations
	Abnormal lung function (obstructive, mixed, restrictive, reduced DL_CO_)	
	Detectable abnormalities on chest imaging such as bronchiectasis, fibronodular scarring	
Severe	Significant and debilitating symptoms that reduce a person’s quality of life and may also affect ability to carry out daily tasks	High risk of future complications such as recurrent chest infections, chronic fungal infection (including aspergillosis) and haemoptysis
	Lung function testing typically shows abnormalities	Increased mortality risk
	Chest imaging typically demonstrates significant structural lung disease such as parenchymal lung destruction, bronchial wall thickening, bronchiectasis and cavitation	

^*^ Based on limited and/or low-quality evidence.

PTLD = post-TB lung disease; DL_CO_ = diffusion capacity of lungs for carbon monoxide.

#### Clinical findings

Complex structural lung disease is often complicated by co-infection with pathogens other than TB. Acute haemoptysis can be a diagnostic challenge for the clinician, as recurrent TB must first be excluded.^110^ A person’s symptoms, clinical presentation, lung function and radiological changes may not always correlate with the extent of structural lung disease.^111^ It is therefore important to perform a baseline assessment for comparison over time.

#### Lung function and imaging

**Figure 5:**
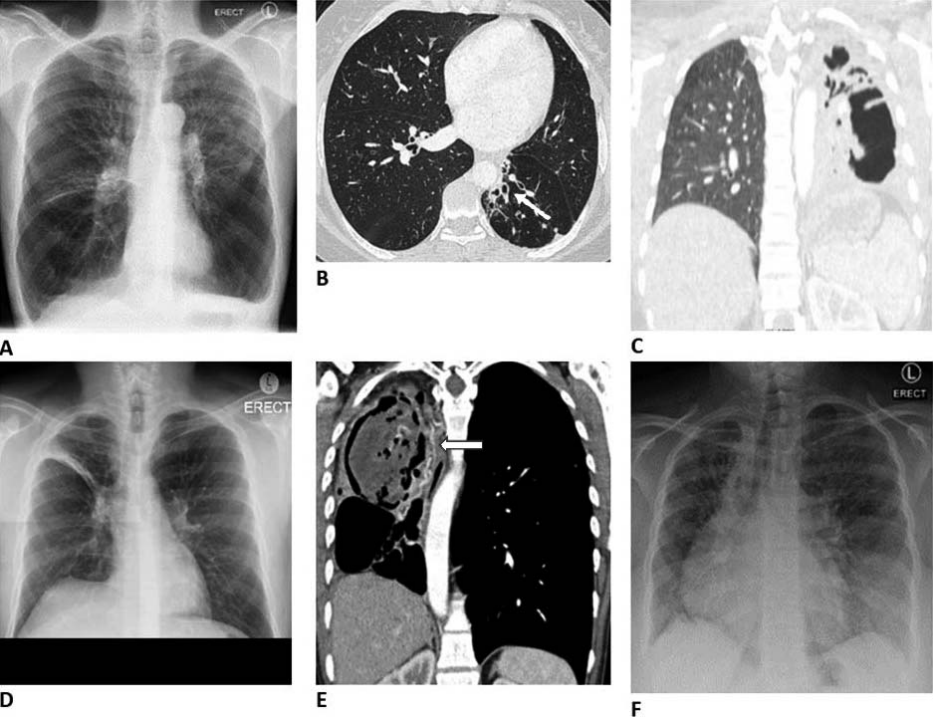
Images and case descriptions corresponding to clinical patterns in post-TB lung disease (see Table 2): A) TOPD in 58-year-old male with three previous episodes of TB, a 10 pack-year history of smoking, and a FEV_1_/FVC of 28%. B) Focal bronchiectasis of the left lower lobe (arrow) in a 38-year-old female with two previous episodes of TB. C) Complete destruction of the left lung in a 31-year-old female, after two episodes of TB. D) Residual fibrotic band in the right upper lobe of a 46-year-old male, with one episode of TB 5 years prior. E) Right upper lobe cavity containing an aspergilloma in a 40-year-old female with recurrent haemoptysis after four previous episodes of TB. Note the dilated bronchial arteries (arrowhead). F) Chest X-ray of a 29-year-old female with pulmonary hypertension after two episodes of drug-resistant TB. Her mean pulmonary artery pressure was 65 mmHg at right heart catheterisation, and she had features of both TOPD and bronchiectasis. Images courtesy of B. Allwood. TOPD = TB-associated obstructive lung disease; FEV_1_ = forced expiratory volume in 1 sec; FVC = forced vital capacity.

Lung function findings are highly variable among people with PTLD (Table 2).^20^,^118^,^119^ Lung volumes may be normal, reduced or increased, while spirometry may show findings diagnostic of obstructive disease, or suggestive of restriction, along with abnormal inspiratory and/or expiratory flow volume loops. Diffusion capacity of carbon monoxide (DL_CO_) may be reduced. Multimodal assessments are necessary because there is a poor correlation between physiology, including lung function, functional capacity and symptoms.^119^ Some people with PTLD demonstrate restriction on lung volume testing because of parenchymal lung destruction, pleural-based abnormalities or chest wall defect. It is possible that severe PTLD could be associated with impaired ventilation during sleep, but robust evidence is absent. Pre-TB baseline spirometry values are rarely known among people who have undergone TB treatment.^5^ An individual’s spirometry results are thus interpreted by comparison with a reference population matched for parameters, including age, sex and height. The absence of abnormalities detected on spirometry or other measures of static lung function does not therefore necessarily indicate that TB caused no impairment of lung function. Loss of lung function due to TB may not be detected if an individual’s spirometry values are considered ‘normal’ because they fall within two standard deviations of the population reference range. Individuals with a history of recurrent TB have a higher risk of lung function impairment due to cumulative lung damage.^110^ The trajectory of lung function loss over time may also be altered by a TB episode. An individual’s loss of lung function over time will not be detected on a single assessment at the completion of TB treatment. This highlights the importance of follow-up post completion of TB treatment and multiple assessments over time. How this is done and by whom should be appropriate to local resources. Triaging those at highest risk after an initial assessment seems prudent. Failure to identify abnormalities on lung function testing or chest imaging could place PTLD survivors at increased risk of further decline by missing opportunities for clinical intervention.^118^ The early recognition of chronic airflow obstruction for example, enables clinicians to minimise further lung insults (e.g., through smoking cessation therapy and vaccination) and initiate treatments that may improve lung function and reduce the frequency of exacerbations. However, few clinical studies have evaluated pharmacotherapy interventions in a PTLD population. For people who smoke, the identification of a measurable lung function abnormality may help motivate them to access smoking cessation interventions and successfully quit smoking. Other abnormalities, such as tracheobronchial stenosis and bronchiectasis, are often under-recognised, particularly when there is limited access to imaging. These can easily be missed with chest X ray or spirometry alone (Figure 5).

**Table 2:** Suggested classification of PTLD clinical patterns^*^

Compartment	Clinical patterns	Definition
Airways	TB-associated obstructive lung disease	Airway obstruction (FEV_1_/FVC ratio <0.7 or <LLN) thought to be primarily related to small airway disease (Figure 5A)
	Tracheobronchial stenosis	Narrowing of the trachea and/or airways, which can increase airway resistance
	Bronchiectasis	CT definition: thickening of airway wall, evidence of airway dilatation > diameter of adjacent vessel, or non-tapering; *OR* CXR definition: evidence of ring shadows and tramlines (Figure 5B)
Parenchyma	Cavitation	A gas-filled space either within an area of pulmonary consolidation, mass or nodule (Figure 5E)
	Parenchymal destruction	Extensive destruction of lung tissue, with a gas-filled space occupying the volume of ≥1 lobe (Figure 5C)
	Fibrotic change	Areas of parenchymal scarring, with associated volume loss (Figure 5D)
	Aspergillus-related lung disease	Evidence of radiological change associated with chronic pulmonary aspergillosis, including pleural thickening, aspergilloma, thin/thick-walled cavities, associated with positive cultures and/or immune assays (Figure 5E)
Pleural	Chronic pleural disease	Evidence of pleural thickening on CXR or CT imaging.
Pulmonary vascular	Pulmonary hypertension	Elevated pulmonary artery pressures as estimated using doppler echocardiography or measured at right heart catheterisation (Figure 5F)
Other	Other	Other pathology, not meeting criteria above

^*^ Adapted from 5.

PTLD = post-TB lung disease; FEV_1_ = forced expiratory volume in one second; FVC = forced vital capacity; LLN = lower limit of normal; CT =computed tomography; CXR = chest X-ray.

### Assessment of PTLD

**Figure 6:**
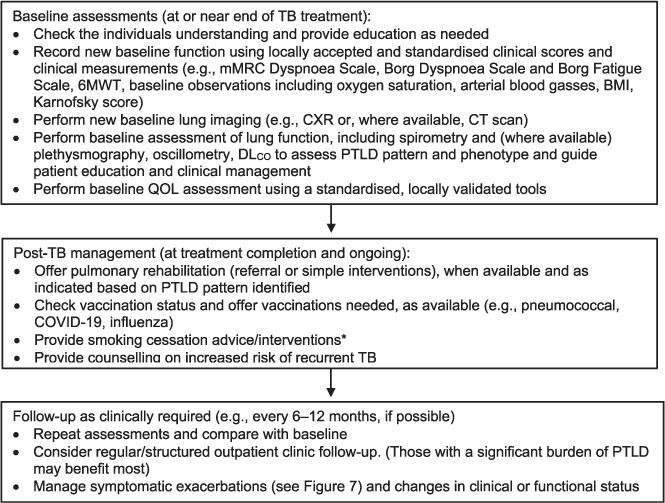
Recommendations for assessment and care planning for TB treatment. A systematic approach to post-TB follow-up is recommended, including a baseline assessment (ideally recorded at, or just before, the end of TB treatment) to allow objective comparison of change over time. *Can be initiated at any time during or after TB treatment. mMRC = Modified Medical Research Council Dyspnoea Scale; 6MWT = 6-minute walk test; BMI = body mass index; CXR = chest X-ray; CPExT =cardiopulmonary exercise test; CT =computed tomography; DL_CO_ = diffusion capacity of lungs for carbon monoxide measurement; PTLD = post-TB lung disease; QoL = quality of life.

The patterns of PTLD are diverse and heterogeneous and may be under-recognised by both people who have had TB and clinicians, particularly if specific screening tests are not performed as routine care at the completion of TB treatment.^5^,^46^ Given the diagnostic challenges in this group, an agreed standardised approach to time-points for clinical assessment would be useful. Although limited prospective data are available, a baseline assessment near completion of TB treatment (after the patient is deemed non-infectious) as a minimum would seem most appropriate.^46^ These results can then be utilised as a ‘baseline’ point of reference for clinical review and appropriate follow-up and individualised interventions can be planned (Figure 6). PTLD and other chronic lung diseases likely have shared risk factors that include exposure to indoor air pollution and smoking. Individuals’ engagement with health care, both during and after TB treatment, presents an opportunity to screen for concurrent lung disease. There is currently no severity scoring system based on individuals’ data that is validated against outcomes. Developing such a tool, applicable in both high- and low-resource settings, is a research priority to allow populations to be stratified according to risk of adverse outcomes.^5^

#### Toolkit for diagnostic assessment

Though a standardised approach to assessment and management is preferable, it may not always be appropriate, given the heterogeneity of disease burden and clinical presentation. There is little evidence to ascertain the optimal combination of diagnostic methods, and how these relate to morbidity and mortality. Further, not all diagnostic methods are available in all settings. The following may be useful in the assessment of PTLD:

- Clinical history: reported symptoms can include dyspnoea, cough, sputum, wheeze, chest pain, haemoptysis, weight loss and fatigue;
- Clinical observations: respiratory rate, heart rate and body mass index;
- Measures of oxygenation and ventilation: resting pulse oximetry, walking oximetry, arterial blood gas, overnight pulse oximetry and diagnostic sleep studies (if indicated and available);
- Assessment of lung function: pre- and post-bronchodilator spirometry, gas transfer (e.g., DL_CO_), body plethysmography for lung volumes, and inspiratory and expiratory pressure;
- Serial lung imaging: CXR or CT (with high-resolution reconstruction, if available) performed at the end of treatment and repeated during long-term monitoring;
- There is also a range of questionnaires and tools (Table 3) that can be used to assess PTLD.

**Table 3:** Tools relevant to the assessment of PTLD

Dyspnoea scales
Modified Medical Research Council Dyspnoea Scale Dyspnoea Scale
Borg Dyspnoea Scale and Borg Fatigue Scale
University of California, San Diego (San Diego, CA, USA) shortness of breath questionnaire
Quality-of-life scales
St George Respiratory Questionnaire
The five-dimension European Quality of Life scale (EuroQol 5)
COPD assessment test
Frailty Index
Karnofsky performance scale
Patient Health Questionnaire (PHQ-9)
Standardised exercise tests
Sit-to-stand test
6-min walk test (6MWT)
Shuttle test (endurance or incremental, depending on the clinical assessment)

PTLD = post-TB lung disease; COPD = chronic obstructive pulmonary disease.

Preferably these should be used in combination to assess an individual’s overall burden of PTLD. For dyspnoea assessment tools and quality-of-life assessment tools, local translation and validation (potentially with local modification) would be vital before they can be effectively applied in a national TB programme.

#### Lung function testing

Measurement of total lung capacity and pre- and post-bronchodilator spirometry may show restrictive, obstructive or normal patterns, despite significant structural lung disease.^118^ Delayed diagnosis and recurrent episodes of PTB are likely to contribute to more significant structural lung damage^111^,^120^ and a decline in lung function.^113^ It would seem reasonable to assume that more severe cavitation and lung destruction involving proportionally more of the lung parenchyma are associated with more severe chronic respiratory symptoms and correlate with more significant lung function impairment. However, symptoms do not always correlate well with disease severity assessed by imaging, spirometry or clinical scores,^111^ and more data are needed on these relationships. Reduced DL_CO_ has been reported to be one of the most frequent abnormalities in PTLD.^118^ It can reflect parenchymal loss, airway disease or pulmonary vascular disorders such as pulmonary hypertension, and is also seen in people who smoke, those with anaemia or hemoglobinopathies, and in people with significant comorbid disease. Quality control is essential when measuring lung function. Standardisation of spirometry is also important for clinical practice and research. The technical statement on standardisation of spirometry by the American Thoracic Society (ATS) and the European Respiratory Society (ERS)^121^ is commonly used. It remains unclear which reference ranges to use for standardisation of spirometry globally, especially in low-income settings with populations of different ethnicities. Options include local or regional guidelines, the Global Lung Initiative multi-ethnic reference values published in 2012 (GLI-2012),^122^ and the Third National Health and Nutrition Examination Survey (NHANES III) reference standards.^123^ There is also currently no consensus on whether absolute percentage predicted cut-offs of lower limit of normal (LLN) values should be used to define abnormal measurements. In the absence of current consensus, clinicians assessing lung function in people who are recovering from TB are advised to consider which approach they will use to assess lung function and apply it consistently.

#### Assessment of exacerbations

**Figure 7:**
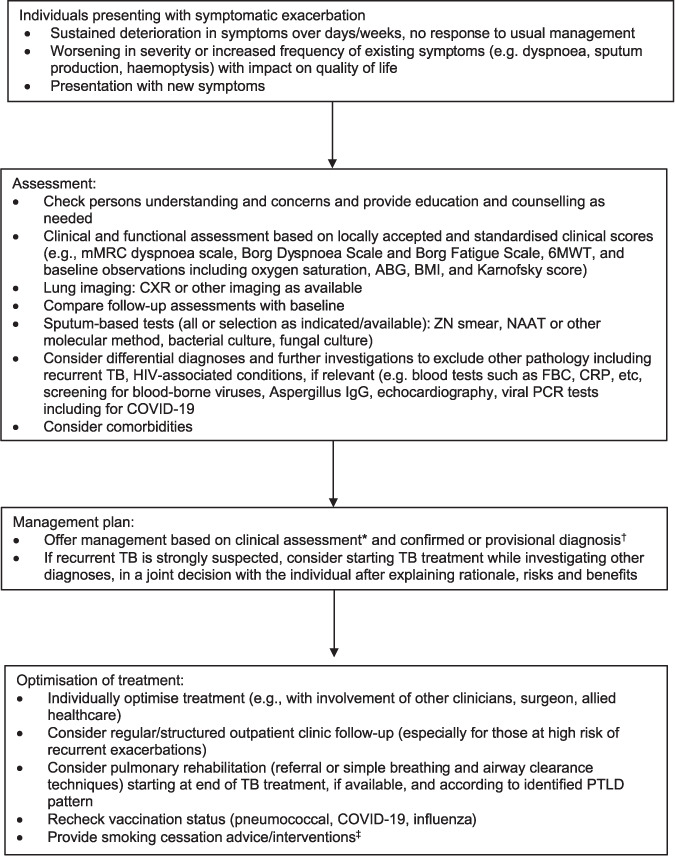
Proposed approach to clinical assessment of symptomatic exacerbations of PTLD. *Lung function tests are generally not required at every clinical assessment or during an acute exacerbation but should be used as a diagnostic investigation and repeated to compare with baseline values when clinical deterioration is observed. ^†^Based on a comparison of assessment findings with baseline assessment at end of TB treatment, and on results of current investigations. ^‡^Can be initiated at any time during treatment or follow-up. mMRC = Modified Medical Research Council; 6MWT = 6-min walk test; ABG = arterial blood gas; BMI = body mass index; CXR = chest X-ray; ZN = Ziehl-Neelsen; NAAT = nucleic acid amplification test; FBC = full blood count; CRP = C-reactive protein; IgG = immunoglobulin G; PCR = polymerase chain reaction; PTLD = post-TB lung disease.

Exacerbations of PTLD can present with a wide range of symptoms, and clinicians must therefore consider a long list of differential diagnoses. The most frequently reported symptoms are non-specific: worsening dyspnoea and/or cough. Chest pain and varying degrees of haemoptysis are also common. Before symptoms can be confidently attributed to a PTLD exacerbation, other diagnoses need to be considered and if necessary, excluded. To note, people with a history of TB are at risk of a recurrence of PTB,^124^ particularly within the first 18 months after treatment. The possibility of recurrent PTB should therefore be considered and investigated in individuals with suspected PTLD. When anti-TB treatment is indicated, it should be initiated as soon as possible to minimise further worsening of PTLD. However, not every exacerbation of PTLD is due to recurrent PTB, and unnecessary courses of ‘empiric’ TB treatment place individuals at significant risk of drug side effects and psychological impairment.^110^,^127^ The difficulty in differentiating PTB from PTLD exacerbations should not be underestimated. Symptom screening and symptom duration alone are not specific enough to distinguish PTLD from recurrent TB and should not be relied upon. A single CXR taken during a suspected PTLD exacerbation can be difficult to interpret in isolation. Ideally, clinical assessment of new or worsening symptoms in a person with a history of PTB, with or without a diagnosis of PTLD, should follow a standardised approach based on the resources available locally (Figure 7).

#### Limitations of TB diagnostic tests for PTLD

Conventional TB diagnostic tests have limited application in people with PTLD. We recommend that clinicians combine multiple diagnostic methods, where available. Access to diagnostics may be limited in many settings.

Nucleic acid amplification tests: Clinicians should be very cautious when interpreting nucleic acid amplification tests including Xpert^®^ MTB/RIF and Xpert^®^ MTB/RIF Ultra (Cepheid, Sunnyvale, CA, USA). The specificity of nucleic acid amplification tests as a test for active TB is reduced in people with a history of treated TB,^125^–^127^ because results may remain positive after completion of successful TB treatment. The rate of false-positive tests is inversely proportional to the time since the person’s most recent episode of TB treatment.^16^,^17^ Higher cycle threshold values have been associated with false positivity,^125^ although not conclusively. In the absence of formal guidelines or expert consensus on the interpretation of these tests in individuals with a history of treated TB, an individual’s results should be interpreted in conjunction with other assessments and diagnostics.

Sputum smear microscopy: Sputum smear remains an accessible first-line TB test in many places. However, clinicians are generally advised not to use sputum smear microscopy as a single diagnostic test. In people with PTLD, a negative smear does not exclude a recurrent episode of PTB, while a positive smear could be indicative of nontuberculous mycobacteria (NTM). Clinical and radiological presentation of infection with NTM can mimic TB. Regardless of the smear result, an alternative microbiological test should be performed (nucleic acid amplification test or culture) to confirm the presence or absence of *Mycobacterium tuberculosis*.

Chest imaging: The specificity of chest imaging, particularly CXR, may be reduced in the assessment of PTLD. Imaging should be used in combination with clinical and microbiological assessments to establish a diagnosis. Images should be compared those obtained at completion of TB treatment to identify new abnormalities.

#### Diagnosis of chronic pulmonary aspergillosis

Chronic pulmonary aspergillosis (CPA) incidence is higher post TB, particularly in people with cavitatory lung disease.^128^ The global burden of CPA is not accurately known because there have been few prevalence surveys, and its epidemiology is probably highly geographically variable. The diagnosis of CPA is predominantly based on a combination of radiological signs and clinical symptoms, supported by microbiological testing and immune testing, if available. Diagnostic guidelines developed by the Infectious Diseases Society of America^128^ and by the European Society for Clinical Microbiology and Infectious Diseases and ERS^129^ are currently recognised internationally.

#### Assessment of recurrent haemoptysis

Haemoptysis in an individual with PTLD could have many underlying causes, ranging in presentation from minimal quantity to life-threatening. Differential diagnosis includes recurrent PTB, acute lower respiratory tract infection (bacterial or viral), bronchial inflammation or hypervascularisation, pulmonary hypertension, cardiac failure (associated with or without PTLD), CPA or other invasive or semi-invasive fungal disease, NTM infection and other potential aetiologies such as diffuse alveolar haemorrhage syndromes. Individuals experiencing haemoptysis may require a combination of chest imaging, sputum microbiological assessment, cardiac examination and assessment by a surgeon and/or interventional radiologist, as appropriate, to identify the cause.

#### Chronic cough and dyspnoea

Chronic cough and dyspnoea may be the main presenting symptom or a predominant symptom in individuals with PTLD. Chronic cough can significantly impair daily functioning, ability to work, quality of life and mood.^3^

#### Comorbidities

The list of potential differential diagnoses associated with PTLD phenotypes or conditions with overlapping presentations is long and challenging (Table 4).^5^ The challenge is compounded by the fact that PTLD most commonly occurs in high TB burden settings where TB will remain one of the most common reasons for respiratory symptoms. There is considerable geographical variation in the epidemiology of these conditions. For many of these comorbidities, diagnosis can be challenging in both high- and low-burden TB settings. All individuals with a current or prior history of TB should be encouraged to undergo HIV testing. Dual pathology should always be considered. The presence of cavitation in those presenting with symptoms post TB should prompt consideration of CPA and testing to exclude NTM. Clinically, PTLD is more frequently not symmetrical and effects the lower lobes disproportionally less. PTLD may be superimposed on another lung disease.^5^ Multiple respiratory exposures and dual pathology are common (e.g., smoking, occupational lung disease). Comorbidities may also influence the risk and trajectory of both PTLD and other respiratory pathology. Specific post-TB parenchymal changes and scarring, including bronchiectasis and post-TB fibro-cavitatory diseases, may put individuals at higher risk of developing CPA and NTM infections.

**Table 4:** Potentially comorbid or alternative diagnoses among individuals with PTLD

Chronic pulmonary aspergillosis NTM infections Chronic colonisation and disease with non-Aspergillus species (e.g., *Pseudomonas*, *Haemophilus*, *Staphylococcus aureus*, *Nocardia*, NTM) Occupational lung disease (e.g., silicosis) Mycoses (e.g., histoplasmosis, cryptosporidiosis, pneumocystis): geographic distribution and epidemiology varies Smoking-related diseases including those associated with exposure to indoor smoke (e.g., emphysema, bronchiectasis) HIV and its complications Pulmonary hypertension Thoracic malignancy COPD Asthma COVID-19 and post-COVID-related lung disease

NTM = non-tuberculous mycobacteria; COPD = chronic obstructive pulmonary disease.

### Management of PTLD

A simple, algorithm-driven approach is preferable, with a focus on syndromic management of respiratory symptoms, adapted from evidence-based guidelines.

#### Management of chronic airway disease

Management will largely depend on the phenotype (e.g., large vs. small airways disease, parenchymal).

Small airways involvement: consider treatment as airway disease/COPD, with a combination of bronchodilators, inhaled or systemic corticosteroids, and antibiotics when indicated.^130^

Large airways involvement: treat as bronchiectasis. Use sputum clearance techniques with antibiotics for infective exacerbations, unless stenotic. For stenotic disease, specific and specialist advice is needed.

Restrictive airway disease: focus on optimising exercise capacity and minimising pulmonary insults. Provide vaccination and treat infective exacerbations.

#### Medical management

There is very little high-quality evidence specific to the medical management of PTLD. In the absence of specific and reliable data, the following recommendations are based on expert opinion informed by evidence from populations with similar phenotypes such as COPD.

Antibiotics: Antibiotic treatment should be reserved for bronchiectatic exacerbations with persistent or worsening symptoms; acute bacterial infection and symptomatic presentation is common. The optimal duration and choice of antibiotics for PTLD exacerbations are not known. Chronic antibiotic use (such as long-term azithromycin) could have additional unwanted consequences such as contributing to antimicrobial resistance. If possible, sputum testing for bacterial, fungal and mycobacterial culture should be performed. Where empiric antibiotic selection is necessary, it should be based on organisms previously cultured in the individual, local guidance and known resistance patterns. Duration of antibiotic treatment, when indicated, should be guided by clinical improvement. A course of between 5 and 14 days is suggested, depending on the individual’s clinical features. In individuals with bronchiectasis phenotypes, baseline sputum production (prior to the exacerbation) should be used as a reference point to determine clinical improvement. Haemoptysis is common during an acute bacterial exacerbation. If the volume is large or haemoptysis recurs, advice should be obtained from a surgeon and interventional radiologist, and they should be referred for assessment for definitive treatment.

Bronchodilators/inhaled therapy: Short-acting bronchodilators for symptom relief are readily available in most settings and supported by COPD guidelines such as those by the Global Initiative for Chronic Obstructive Lung Disease.^130^ The use of long-acting beta_2_ agonists (LABA) or long-acting muscarinic agonists (LAMA) for person with PTLD with post-TB obstructive airway disease (small airways disease), may be beneficial and should be considered on a case-by-case basis. After starting inhaled treatment, their effectiveness in reducing exacerbation frequency and symptoms should be assessed. Evidence is needed on their effects on lung function, exacerbation rates, hospitalisation rates and mortality rates.

Systemic corticosteroids: There is limited evidence for the use of oral corticosteroids in exacerbations of PTLD. However, corticosteroids are commonly used in the management of exacerbations of asthma and COPD or as part of their management in many settings. Caution should be taken with the use of repeated courses, as there is evidence that these can contribute to immunosuppression and reactivation of TB.^131^

Inhaled corticosteroids: The safety and efficacy of inhaled corticosteroids in the management PTLD are not yet known. There are concerns about their potential to increase the risk of NTM infection^132^,^133^ or reactivation of TB.^134^,^135^ In the absence of PTLD-specific safety data, we recommend caution in the use of inhaled corticosteroids in individuals with PTLD, with frequent reassessment after starting treatment, unless otherwise indicated (for example, in the treatment of asthma)

Antifungal agents for CPA: Few prospective studies have evaluated the effectiveness of oral antifungal agents for CPA, or identified which individual patients would benefit most or the optimal duration of treatment. Azole therapy (usually oral itrazonazole at 400 mg/day) given for 6 months or longer^136^ is often first-line treatment of choice for those with aspergilloma or chronic cavitatory-type presentations of CPA, with or without haemoptysis; in case of the latter, it is sometimes given alongside tranexamic acid.^129^ Aspergillus immunoglobulin G antibody titres can be a useful additional test to help guide treatment decisions. The benefit of triazole antifungal agents such as itraconazole in the treatment of CPA in persons with PTLD is uncertain.^137^ These agents and aspergillus-specific tests are not widely available in all settings; the treatment is expensive and would be required for prolonged periods in this group of individuals, with some requiring repeated courses of treatment. Azole resistance has been reported and the benefit of triazole anti-fungal agents in the treatment of PTLD is uncertain.^137^ It should be noted that triazole antifungals and anti-TB therapy cannot be given concurrently due to their potent drug–drug interactions.

#### Surgical treatment and bronchial artery embolisation

Although there is evidence to support the role of targeted thoracic surgery as an adjunct to chemo-therapy in the context of MDR-TB,^138^ few studies have evaluated the role of surgery for the specific management of symptomatic PTLD. Surgical resection can be recommended in some individuals with PTLD who have recurrent massive haemoptysis or recurrent exacerbations with significant focal lung destruction. However, these presentations are relatively rare outside of specialised referral centres. Surgical resection can only be considered when the burden of disease is confined to an anatomically resectable area of the lung, and the person has adequate physiological reserves to tolerate lung resection. Furthermore, the surgical and support skills required to perform these operations are not always available, and careful consideration of the risks and potential benefits needs to be made before proceeding in an individual. Assessment of individuals for lung resection surgery is beyond the scope of this guideline. When available, and for massive or recurrent haemoptysis, bronchial artery embolisation can reduce the rate of recurrence of haemoptysis episodes, particularly in surgically irresectable disease.^104^

#### Pulmonary rehabilitation

Much of the evidence of benefit for pulmonary rehabilitation is derived from studies in patient populations with other chronic lung diseases, predominantly obstructive and bronchiectatic disease. Evidence specific to PTLD is emerging, but more is needed to ascertain its effects.^139^ Preliminary data from small studies show that pulmonary rehabilitation programmes can be beneficial for dyspnoeic PTLD individuals with impaired lung function. Access to pulmonary rehabilitation programmes in high TB burden settings is currently limited, and more data are needed to understand the best combination of tools and techniques that could be both beneficial and widely accessed by providers and individuals.^140^ Pulmonary rehabilitation studies in persons with PTLD have mainly used a holistic approach, incorporating techniques including the 6-min walk test, breath holdings and breathing exercises, and airway clearance, with dietary advice and psychological support.

### Tertiary prevention of TB

Prevention of recurrence involves a broad range of multi-level individual and community public health interventions focused on earlier diagnosis and prevention of household transmission. These include screening of household contacts, including management of latent TB infection, education and awareness to encourage early clinical presentation, nutrition and economic support.^124^

#### Vaccination

There is limited evidence from prospective studies assessing outcomes of vaccination in individuals with PTLD. Immunisation against influenza, COVID-19 and pneumococcal disease is recommended, based on existing evidence from studies of individuals with other chronic lung diseases.

#### Smoking cessation interventions

Studies show that people who continue smoking tobacco throughout TB treatment have worse outcomes and higher rates of recurrence later.^140^ Smoking cessation interventions should therefore start early in treatment for the best chance of success. However, smoking cessation at any point in the treatment course, including at end of TB treatment, may still have major benefits in reducing risk of recurrence and preservation of lung function. The Union’s 2010 guidelines for smoking cessation and smoke-free environments for those diagnosed with TB recommend the ‘ABC for TB’ (Ask, Brief advice, Cessation support) approach at each health-care visit.^141^ Smoking cessation interventions can include nicotine replacement therapy and a range of psychological support delivered by clinical or trained nonclinical staff.

## 3. POST-TB CARDIOVASCULAR AND PERICARDIAL DISEASE

People with active TB and TB survivors can experience various manifestations of cardiac disease, including pericardial and myocardial disease, valvular disease and rhythm disturbances.^142^–^145^ This clinical statement does not cover rarer manifestations of TB-associated cardiac disease, for which there is limited evidence to guide management. Cardiovascular disease (CVD) here refers to coronary artery disease (myocardial infarction, acute coronary syndromes, coronary death and chronic heart failure), cerebrovascular disease (stroke and transient ischaemic attack) and peripheral artery disease.

### Tuberculous pericarditis

Tuberculous pericarditis is the most common cardiac presentation of TB, affecting approximately 1–2% of people with TB, with higher incidence in people living with HIV.^146^–^148^ During treatment, TB pericarditis is associated with high mortality, ranging from 8% to 17%, among people uninfected by HIV, but increasing to 40% in people living with HIV.^149^–^151^ Tuberculous pericarditis often presents insidiously, with non-specific respiratory or constitutional symptoms, including cough, dyspnoea, chest pain, night sweats, fatigue and weight loss, which may result in a delayed or missed diagnosis. Clinical signs typical of pericarditis, cardiac tamponade or heart failure may also be present.^149^,^152^,^153^ Constrictive pericarditis is the most significant chronic sequelae in tuberculous pericarditis and may occur in up to 30–60% of people with TB pericarditis, despite TB treatment and use of corticosteroids.^152^–^154^

### Cardiovascular disease in people with TB

People with TB have increased risk of myocardial infarction, acute coronary syndrome, ischaemic stroke and peripheral artery disease, and long-term CVD mortality.^71^,^155^–^158^

#### Risk factors for cardiovascular disease post TB

Traditional CVD risk factors, which include smoking, DM, hypertension and dyslipidaemia, are likely to be key drivers of CVD outcomes in this group of people. Smoking and DM are associated with increased incidence of TB, and are therefore common comorbidities among individuals with TB.^159^–^161^ People living with HIV, which is a major risk factor for TB, also have elevated CVD risk after adjusting for traditional CVD risk factors.^162^,^163^ The underlying mechanisms of increased CVD risk in TB infection and disease are not well understood, but some studies suggest that TB may lead to CVD through immune activation, autoimmunity or a direct effect on the heart and vasculature.^155^,^156^,^164^,^165^ In addition, CVD and TB disease are both strongly associated with socio-economic status. Populations with the highest risk of TB may also have high risk of CVD due to exposure to environmental and socio-economic risk factors common to both conditions over their life course.^166^ To our knowledge, no distinct TB CVD clinical phenotypes have been identified.

#### Cardiovascular risk stratification

CVD risk stratification is conventionally used to guide primary CVD prevention in the general population. It is possible that CVD risk prediction tools used in the general population, such as the SCORE model,^167^ Framingham score,^168^ or the American College of Cardiology/American Heart Association (ACC/AHA) atherosclerotic CVD risk calculator (ASCVD)^169^ may under-estimate CVD risk among TB survivors. A recent study found that latent TB infection was independently associated with an increase in the odds of subclinical obstructive coronary artery disease, even after adjusting for 10-year ASCVD risk score and HIV status.^170^ No CVD risk prediction tools have been validated for use in TB survivors, and the absolute and attributable risk associated with each risk factor has not been described among TB survivors.

### Clinical management

#### Management of tuberculous pericarditis

Early identification of tuberculous pericarditis and prompt initiation of anti-TB therapy are essential. Other therapies, including the use of corticosteroids, are more controversial. The WHO evaluated the use of corticosteroids in the management of tuberculous pericarditis for the 2017 drug-susceptible TB guidelines, and noted very low certainty of evidence based on the GRADE (Grading of Recommendations, Assessment, Development and Evaluations) framework.^171^ We support the WHO guideline’s conditional recommendation that corticosteroids be used as adjunctive treatment for individuals with tuberculous pericarditis.^171^ Chronic constrictive pericarditis is responsible for most longer-term morbidity and mortality in people with tuberculous pericarditis. Prompt TB treatment is the key strategy to reduce complications related to chronic constrictive pericarditis.^172^ In individuals with persistent symptomatic constrictive pericarditis, pericardiectomy should be considered even in case of adequate TB treatment; however, optimal timing is unclear.^152^,^172^,^173^

#### Cardiovascular risk management

In the absence of robust evidence to inform CVD risk assessment and management among TB survivors, we advocate a pragmatic approach that addresses both traditional CVD risk factors and other modifiable factors such as HIV treatment (Table 5). We suggest the use of quantitative CVD risk prediction tools to estimate CVD risk.^169^ Available data suggest that increased CVD risk is observed among TB survivors for over a decade after TB treatment completion. This prolonged timeline offers an opportunity to improve outcomes after TB therapy and highlights the need for long-term management strategies for risk factors, and coordinated efforts between TB programmes, primary care and non-communicable disease programmes.^156^ Risk factor modification with non-pharmacologic and pharmacologic interventions should be considered for people with TB disease and TB survivors, including adequate management of blood pressure, dyslipidaemia and DM, as well as interventions to reduce smoking, increase physical activity, reduce obesity and improve diets. The choice of interventions should be based on the balance of risks and benefits and be suitable to the regional context. Low-cost, low-risk lifestyle interventions can be recommended for all. Pharmacotherapies are more costly for health systems, may contribute to the pill burden, may be associated with adverse effects and may require individuals to spend time and money on regular healthcare. Given these considerations, we recommend that TB programmes consider pharmacotherapies in the setting of established primary care programmes to ensure that treatment is safe, effective and sustainable for years after TB treatment completion.

**Table 5:** Suggested approach to cardiovascular risk factor modification in people with TB

Risk factor	Guidelines	Intervention	Approaches
Smoking	Smoking cessation and smokefree environments for TB patients (The Union, 2010)^*^	Screening	**A**sk: all individuals at each healthcare visit if they currently smoke and if anyone smokes inside their home
		Smoking cessation	**B**rief Advice: at each visit advise people with TB and TB survivors to quit smoking or to continue not to smoke (see Table 2.1, The Union 2010^141^)
			**C**essation support: practical help with planning to quit smoking and making the home smoke-free; ask about previous attempts to quit, advise complete avoidance, explain harms, advise on coping with nicotine withdrawal and weight gain, nicotine replacement therapy and other medicines, if available (see Table 2.1, The Union 2010^141^)
Diabetes mellitus	Management of DM-TB: a guide to the essential practice (The Union, 2019)^161^	Screening	Recommended: screening for hyperglycaemia at TB diagnosis in all individuals not known to have DM (targeted screening based on local epidemiology can be used in low-resource settings)
			Suggested screening approach: blood glucose on opportunistic sample. If positive, FBG/HbA1c to confirm
			Note: Retesting at TB treatment completion is useful to confirm the diagnosis of DM and inform future management
		Glycaemic control	Aim to achieve glycaemic control during TB treatment, with management within the TB clinic for the first 2–8 weeks;
			Target: HbA1c <8% or FBG <10 mmol/L (180 mg/dL) during TB treatment;
			For people with previous diagnosis of DM, assess glycaemic control with FBG or HbA1c;
			Recommended for all: lifestyle and dietary advice;
			Pharmacological management if required; suggested approach (in absence of regional guidance): Metformin If target not reached, add sulphonylurea (note 30–80% reduced efficacy with rifampicin) If target not reached, change to insulin
Dyslipidaemia	Regional guidelines^*^^169^ for ASCVD prevention	Screening	Suggest screening at sex and age-appropriate cut-offs according to regional CVD prevention guidelines^†^^163^
	Regional guidelines for CVD management	Lipid control	Lifestyle advice: diet, smoking cessation, physical activity and weight management;
			Use of pharmacologic agents following regional guidelines for those with high estimated CVD risk
Hypertension	Regional guidelines^*^^169^	Screening	Suggest screening for hypertension according to regional guidelines
	Regional guidelines	Blood pressure control	Lifestyle advice – diet, physical activity, weight management, and reduced salt intake;
			Use of pharmacologic agents per regional guidelines for persons with high estimated CVD risk

^*^ For more information on the use of quantitative CVD risk predictors in primary care, see Lloyd-Jones DM, et al.^169^

^†^ For more information on CVD risk in people living with HIV, see Feinstein MJ, et al.^163^

DM = diabetes mellitus; FBG = fasting blood glucose; HbA1c = glyclated haemoglobin; ASCVD = atherosclerotic CVD; CVD = cardiovascular disease.

#### Tobacco smoking

Tobacco use is associated with increased TB disease severity, worse treatment outcomes and disease recurrence.^159^,^160^ We support the ‘ABC for TB’ approach recommended by The Union^141^ to help people with TB give up smoking (see Smoking cessation interventions, above).

#### Diabetes mellitus

There is a strong link between DM and adverse TB treatment outcomes.^161^ Updated guidelines for the integrated management of DM and TB, published by The Union in 2019,^161^ recommend screening for DM for all individuals with TB at the time of diagnosis.

We endorse The Union’s recommendation that DM management should be provided within the TB services for the first 2–8 weeks of TB treatment. Given that active TB compromises glycaemic control, the guidelines offer more lenient targets than those recommended in mainstream DM guidelines, including a glycated haemoglobin (HbA1c) target of <8% (compared with the usual target of <7%).^161^ Retesting of blood glucose may be useful to confirm the diagnosis at TB treatment completion.

#### Other comorbidities and chronic disease risk factors

There are no clinical management guidelines specific to the management of hypertension, dyslipidaemia or overweight/obesity in people with TB. Lifestyle modification, including advice about physical activity, weight control and diet, should be routinely recommended for all. Dietary advice will vary according to the local context. Guidelines developed by the American College of Cardiology and the American Heart Association^174^ advise regular intake of vegetables, fruits, legumes, healthy protein sources (fish/seafood, nuts, low fat poultry, low fat dairy), and whole grains, and limiting intake of sweets, sugar-sweetened and artificially sweetened beverages and red meats. Salt intake may be a driver of hypertension in many settings and should be reduced.^174^ All dietary advice given during TB therapy should consider individual resources and food security, and the potential need for nutritional supplementation during TB therapy, with particular consideration of local availability of food and nutrition and socio-economic context.^175^ Advice to individuals on exercise and weight control may vary over the course of TB therapy, given the high caloric needs and disability that people may experience during TB therapy. There is no clear evidence supporting a specific approach to the pharmacological management of blood pressure or blood lipids in people with TB or TB survivors. Similarly, there are no specific evidence-based guidelines for the management of chronic heart failure in people with TB or TB survivors. We suggest that blood pressure and dyslipidaemia should be managed by following regional or international guidelines for the general population, such as those developed by the American College of Cardiology and American Heart Association.^176^

## 4. POST-TB NEUROLOGICAL DISABILITY

Central nervous system (CNS) TB is a devastating form of TB, resulting in death or disability in the majority of affected indidviduals.^177^ Tuberculous meningitis (TBM), and other less common neurological manifestations such as intracranial tuberculoma/abscess and spinal TB, may occur either in isolation or in various combinations. The major intracranial pathological consequences of the inflammatory reaction elicited by *M. tuberculosis* include 1) arachnoiditis, which may result in cranial nerve palsies and obstruction of cerebrospinal fluid flow, 2) vasculitis, which may result in strokes, and 3) tuberculoma/abscess formation. Spinal involvement includes vertebral disease (bony spinal TB), which may result in compression of neural tissues and radiculomyelitis. Neurological TB may be further complicated by cystic cavitation of the spinal cord (syringomyelia), either during or after completion of TB treatment.

### Clinical presentation of post-TB neurological disease

Neurocognitive dysfunction and functional impairment are common complications of neurological TB during the chronic treatment phase and after treatment completion at 6–12 months’ follow-up. A recent systematic review and meta-analysis of outcomes in adults with TBM reported physical disability (defined as any disability that impedes the patient’s ability to carry out tasks they once performed) in 32% (95% confidence interval 22–43) of indidvuals.^178^ Other studies in adult survivors of TBM report severe disability in 14–16% of participants at 9 months’ follow-up^179^,^180^ and in 14% at 5 years’ follow-up.^181^ Other CNS manifestations of TB are also associated with significant long-term disability. Among cohorts of adults with spinal TB (bony and non-bony) followed up for 9 months,^182^ and of people who developed TB-associated syringomyelia,^183^ the majority were unable to walk at the end of follow-up. A study from the same referral hospital further found that only 20 of 54 (37%) adults with intracranial tuberculoma fully recovered at 18 months after presentation.^184^ Neurological sequelae in CNS TB include motor deficits (e.g., hemiparesis or paraparesis),^179^,^185^–^188^ cranial nerve palsies (e.g., vision and hearing impairments),^185^–^187^,^189^,^190^ seizures^191^,^192^ and hydrocephalus, which may require ventriculo-peritoneal shunt insertion.^187^,^188^,^193^ Pain syndromes, such as persistent headaches^194^,^195^ and neuropathic and musculoskeletal pain frequently contribute to disability. Bladder dysfunction is common in people with TBM, particularly if lumbosacral arachnoiditis co-exists; abnormal urodynamic study findings may persist in up to 28% of affected individuals at 6 months’ follow-up.^196^ Adverse events due to TB treatment may also contribute to residual neurological disabilities. These include peripheral neuropathy due to isoniazid treatment, optic neuropathy secondary to ethambutol treatment and hearing loss due to aminoglycosides.^197^ Data on the extent of the neuropsychological impact of TBM in adults are scant, but its consequences are likely to be substantial.^198^ A cohort study from India reported that 55% of patients were found to be cognitively impaired at 12 months’ follow-up using the Mini Mental State Examination.^186^ Two small (*n* = 17, *n* = 19) studies of adult survivors of TBM reported impairment in multiple cognitive domains in patients, compared with healthy controls, when detailed cognitive assessments were performed after treatment completion.^199^,^200^ Another recent prospective study of 60 adults with TBM reported varying degrees of cognitive impairment in more than 90% of 43 patients tested at 1 year follow-up.^201^

### Management of post-TB neurological disease

Despite clear evidence that long-term neurological disability is very common after TBM, standardised evidence-based management strategies have not been developed.

#### Research gaps

The lack of clinical practice guidelines for the management of TBM is partly due to inconsistent outcome reporting across studies evaluating treatment approaches, which prevents comparisons and meta-analysis.^178^,^202^ The Tuberculous Meningitis International Research Consortium recommends that uniform methods to report disabilities should be employed in all TBM studies.^202^ The Modified Rankin Scale should ideally be recorded at 12 months from treatment initiation in all children and adults, and detailed neurocognitive and psychiatric outcomes should be reported, where possible.

#### Principles for clinical management post-TB neurological disease

**Figure 8:**
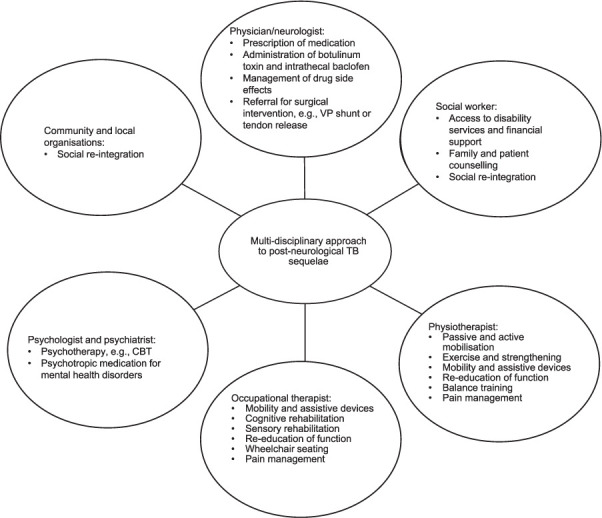
Multidisciplinary team care for people with post-TB neurological disability. VP = ventriculoperitoneal; CBT = cognitive-behavioural therapy.

Management of the long-term neurological sequalae of TB should be based on the general principles applicable to the care of people living with chronic neurological disease. Management should be tailored according to resource availability, as well as the individual’s specific needs. Comprehensive assessment of an individuals’ biopsychosocial functioning and contextual factors would help guide appropriate management planning.^203^ Management strategies should encompass input from a multidisciplinary team (Figure 8) and incorporate adjustments to the individual’s living environment, as well as pharmacotherapy (Table 6). The goal of treatment is to achieve the best possible functional performance, with social reintegration, good mental health and optimal long-term quality of life.

**Table 6:** Pharmacotherapy for neurological sequelae of tuberculous meningitis

Symptom	Possible cause	Recommended medication
Spasticity	Intracranial TB Spinal TB with cord involvement	First-line: baclofen, gabapentin or tizanidine Second-line: diazepam, dantrolene (doses limited by toxicity); intrathecal baclofen pump in wheelchair- or bed-bound individuals with severe spasticity
Persistent headache	Intracranial TB Mental health disorders Primary headache disorders	Considered and limited use of simple analgesia for more severe headaches (e.g., paracetamol or NSAIDs twice a week) Prophylaxis tailored to individual comorbidities: Propranolol 40–80 mg twice daily Sodium valproate/valproic acid^*^ 300–450 mg twice daily Amitriptyline 10–75 mg every night Topiramate^*^ 25–100 mg twice daily Venlafaxine 75–225 mg once daily
Neuropathic pain	Peripheral neuropathy (disease- or drug-induced)	Pyridoxine 25 mg daily to prevent drug toxicity in individuals receiving isoniazid First-line: Pregabalin, duloxetine, gabapentin Alpha-lipoic acid Second-line: Tricyclic anti-depressants Venlafaxine Sodium valproate/valproic acid^*^
Bladder dysfunction	Spinal TB with cord/root involvement Brainstem TB	Failure to store: anticholinergics, e.g., oxybutinin, propiverine, tolterodine, darifenacin, trospium chloride Failure to empty: alpha blockers (e.g., tamsulosin, alfuzosin, doxazosin, silodosin, terazosin)
Bowel dysfunction	Spinal TB with cord/root involvement Immobility from hemi-, para- or quadriparesis Inadequate oral intake due to dysphagia or poor diet	Bulking agents (e.g., sterculia) tool softeners (e.g., lactulose, magnesium sulphate) Stimulant or osmotic laxatives (e.g., Senna, bisacodyl sodium picosulphate, polyethylene glycol with electrolytes)
Sexual dysfunction	Spinal TB with cord/root involvement Mental health disorders (e.g., depression)	Phosphodiesterase inhibitors for erectile dysfunction (e.g., sildenafil, tadalafil, vardenafil)
Pseudobulbar affect	Intracranial TB including bilateral hemispheric or upper brainstem involvement	Dextromethorphan/quinidine 1 tablet twice daily Tricyclic antidepressant, SSRI or SNRI
Seizures	Intracranial TB	Monotherapy (avoid cytochrome P450 inducers to minimise drug–drug interactions) First-line: Lamotrigine Levetiracetam Sodium valproate/valproic acid^*^ Second-line: Carbamazepine or oxcarbazepine Phenytoin ^†^ Adjunctive: Clobazam or clonazepam Pregabalin or topiramate
Cognitive impairment	Intracranial TB	No current evidence for anticholinesterase inhibitors

^*^ Contraindicated in females of child-bearing age, unless using reliable contraception.

^†^ Agents that are inducers of hepatic cytochrome P450 microsomal enzymes are not first-line due to increased risk of drug interactions with anti-TB therapy and antiretroviral medication.

NSAID = non-steroidal anti-inflammatory drug; SNRI = serotonin-norepinephrine reuptake inhibitor; SSRI = selective serotonin reuptake inhibitor.

#### Motor deficits and associated complications from immobility

Residual weakness following TBM is often complicated by spasticity, spasms, tendon shortening and joint contractures. Early involvement of physiotherapists and occupational therapists in the care of affected individuals is essential to achieve optimal long-term motor outcomes. Splinting of affected limbs and muscle strengthening exercises improve functioning and prevent contractures. Passive and/or active stretching, exercises and positioning can reduce spasticity and maintain range of motion of joints. Oral medications may also be effective in reducing spasticity and associated symptoms (Table 6).^204^ In individuals with severe spasticity or joint restrictions, botulinum injections or surgical tendon release, as well as serial casting or splinting should be considered.^205^ Assistive devices such as splints and ankle foot orthoses, commodes, shower chairs and rails may improve the persons mobility or functional limitations of activities of daily living. Immobility itself leads to additional complications such as pressure sores, musculoskeletal pain and an increased risk to develop deep vein thrombosis. The primary preventative measure for these complications is education on self-maintenance of joint range of motion and skin care.^206^ Sufficient cushioning of bony prominences and wheelchair seating by trained rehabilitation staff should be initiated early. Appropriate wound care and analgesia should be implemented for pressure sores and generalised body pain due to immobility.

#### Sensory dysfunction

Complete or partial sensory loss, either tactile (skin) or proprioceptive (joint position sense) in the limbs may result in, or contribute to, poor balance, difficulty walking and increased fall risk. Poor hand function and eye-hand coordination will further limit activities of daily living such as washing and dressing. Each individual should be assessed and then educated about the risk of skin damage and falls. Ideally, independent rehabilitation of balance and proprioception should be attempted, but the use of mobility assistive devices, such as walking aids and wheel-chairs, may be prioritised if safety could be compromised. Devices such as weighted utensils may improve hand function and other activities requiring the use of the upper limbs.

#### Visual impairment

Individuals still receiving TB treatment (particularly an ethambutol-containing regimen) should be instructed to report any visual changes to their health-care provider. Ethambutol should be withdrawn if ethambutol-induced optic neuropathy is suspected. If visual impairment has been identified, diagnostic evaluation of the nature of visual impairment will inform the type of intervention needed. Targeted measures in individuals with partial visual deficits could improve reading ability, orientation and enhance the individuals independence and quality of life.^207^ Central visual field defects may impair reading ability, which could be improved with eccentric fixation and the use of visual aids to magnify the text. Reading training could further improve reading speed. People with concentric or peripheral visual field defects that impair orientation may benefit from mobility training and the use of tactile aids, such as a cane. Many countries have national societies or support organisations for people with blindness. These organisations may aid access to care or rehabilitation professionals and facilitate re-integration back to society, and may provide counselling, education and employment opportunities that assists individuals to cope and live with a new deficit.

#### Hearing impairment

Individuals still receiving TB treatment, particularly regimens containing ototoxic drugs (such as amino-glycosides) should undergo regular screening using audiograms to identify early hearing impairment, which, if present, should prompt discontinuation of the ototoxic agent.^208^ Individuals who present with hearing difficulty require referral to an audiologist, who should institute appropriate interventions such as the provision of a hearing aid or referral for cochlear implants. However, cochlear implants and rehabilitation may be costly and not possible in many LMICs due to limited financial, infrastructure and human resources. Support organisations for people with deafness may aid access to care or rehabilitation professionals and may provide counselling, education and employment opportunities.

#### Nutrition and dietary intake

The causes of inadequate dietary intake in individuals recovering from neurological TB include dysphagia, generalised weakness, medication side effects, pain, hiccups, and, in some instances, refusal of food and medication.^209^,^210^ Initial investigations for dysphagia may include oesophagogastroscopy, barium swallow, fibreoptic bronchoscopy and, CT scan of the chest.^210^ Management of dysphagia encompasses nasogastric tube feeding, and percutaneous endoscopic gastrostomy (PEG) feeding. Nasogastric tube feeding is suitable for short-term nutritional support and PEG feeding is a safe, effective, and acceptable method of providing more long-term enteral nutrition.^211^ It is important to be mindful of cultural values or beliefs and the level of individuals’ or caregivers’ education level and health literacy when PEG is considered.^212^ Appropriate education on PEG care and use should be provided to individuals and their caregivers by trained healthcare workers.

#### Headache and other chronic pain syndromes

Headaches in a people recovering from neurological TB should prompt exclusion of an underlying cause, particularly in HIV-infected persons and those with VP shunts. When appropriate investigations have excluded a secondary cause, the treatment approach is similar to that of any non-specific chronic headache (Table 6).^213^ Non-pharmacological interventions include cognitive-behavioural therapy (CBT) and stress management. Use of simple and combination analgesia needs to be quantified to identify potential medication overuse, which commonly contributes to chronic daily headaches. Management of medication overuse headache includes education, treatment of comorbid depression and anxiety, lifestyle changes and tapering of simple analgesics. Neuropathic pain may impact substantially on daily function and quality of life.^214^ In addition to drug therapy (Table 6), important management strategies include education about chronic pain and counselling on self-directed strategies, such as stress management, along with frequent exercise and CBT.^215^–^217^ Individuals with refractory pain should be referred to a multi-disciplinary pain clinic (if available). Desensitisation,^218^ mirror box therapy, neural mobilisation techniques, dry needling and electrotherapy could provide additional pain relief.^219^ Musculoskeletal pain due to immobility or the involvement of a vertebral body or joint is typically managed with exercises and mobilisation (neural, soft tissue and joint).^220^ However, manual spinal mobilisation is contraindicated in those with spinal TB and an unstable vertebral column. Interventions for management of chronic pain, such as hydrotherapy, transcutaneous electrical nerve stimulation and dry needling could prove beneficial.^216^ Management using a multidisciplinary approach to education and empowerment should also be considered to address the consequences of living a life with chronic pain.^221^

#### Neurogenic bladder dysfunction

The goals of effective bladder management are to prevent urinary tract infections, as well as other complications which may arise from obstruction of the urinary tract, such as hydronephrosis and calculi. The first step in management of neurogenic bladder dysfunction is to assess the individual’s ability to self-catheterise and their level of mobility. Urodynamic studies can categorise the dysfunction into either failure to store, or failure to empty the bladder. Bladder drainage can be achieved with various forms of catheterisation. Failure to store can be managed with a combination of timed voiding, pelvic floor exercises and oral medications (Table 6). Failure to empty may be improved with Valsalva manoeuvres or suprapubic tapping, and medication (Table 6). Surgical options include augmentation cystoplasty, neurostimulation and bladder denervation.^222^

#### Neurogenic bowel dysfunction

Clinical features of a neurogenic bowel dysfunction are constipation, faecal incontinence and abdominal pain. Complications include faecal impaction, ileus, autonomic dysreflexia and haemorrhoids. The initial assessment includes taking a history of diet and symptoms, performing a physical examination and obtaining an abdominal X-ray to look for faecal loading. A basic bowel management programme consists of optimisation of diet and fluid intake, increasing physical activity, optimising positioning and performing manual evacuation of stool and medication (Table 6). Surgical options such as a colostomy or ileostomy may be considered.^223^

#### Post-TB sexual dysfunction

The cognitive and psychiatric sequelae of CNS TB or post-TB neurological disability, as well as new socioeconomic stressors such a loss of employment due to disability, can play an enormous role in sexual dysfunction. These factors need to be identified and addressed. Most of the available literature is focused on the sequelae and management of the male sexual response, and there is relatively little research on the female sexual response in spinal disease. Medical management is limited to the treatment of erectile dysfunction (Table 6).^222^

#### Seizures

In the acute phase of TBM, symptomatic seizures may occur and can be treated in the hospital setting. Some individuals subsequently develop epilepsy and will need long-term anti-epileptic therapy. The choice of anti-epileptic agent needs to take into consideration concomitant anti-TB therapy, as well as other medications such as antiretroviral therapy, to minimise drug interactions (Table 6).

#### Cognitive impairment

The cognitive sequelae of CNS TB are often under-recognised. All individuals would benefit from a detailed neuropsychological assessment to determine the presence, nature and severity of impairment. Assessment should be followed by a multidisciplinary treatment approach, which should consider the individual’s functional, participation and/or social roles in their home, work and community environments. Interventions include counselling and referral to a social worker, occupational therapist and psychologist. Local organisations may provide sheltered work opportunities and other assistance for those with acquired cognitive impairment.

## 5. CHILDREN AND ADOLESCENTS POST-TB DISEASE

### PTLD in children and adolescents

#### Clinical presentations of PTLD in children and adolescents

Data on PTLD in children and adolescents are limited. Quaife et al. estimated that PTLD accounts for approximately half of lifetime DALYs caused by incident TB.^66^ This means that in young children, the life-time impact of PTLD could be even larger than in adults, in combination with the fact that lungs continue to develop until early adulthood, with the first years of life being most critical in lung development.^224^ Despite the lack of data on the long-term impact of PTB on lung health in children, there is clear evidence that lower respiratory tract infections in the early years reduce lung function later in life.^225^,^226^ PTB occurring in a young child or adolescent could therefore have a substantial impact on long-term lung health outcomes. It is also likely that the long-term effects of PTB in children and adolescents depend on the type (parenchymal vs. nodal airway disease or other), the severity and the age of TB onset, which is relevant to age-related lung development.

#### Assessment of PTLD in children and adolescents

End-of-treatment functional and structural assessment enables identification of children at risk of PTLD. Clinical standards for the assessment, management and rehabilitation of post-TB lung disease recommend standard assessment at the end of treatment in children with more severe forms of PTB or in children who remain symptomatic at the end of their treatment (Table 7).^46^ The WHO defines non-severe PTB as smear-negative TB with CXR features that include PTB confined to less than one lobe with no cavities (<1 lobe), no signs of miliary TB, no complex pleural effusion and intrathoracic lymph node TB without significant airway obstruction or bilateral airway narrowing.^227^,^228^ Children with presumed or confirmed drug-susceptible non-severe TB can receive the WHO-recommended shorter TB treatment duration (4 months) and would generally not require assessment for PTLD. However, to date there is no clinical data available to confirm this. Adolescents are more likely than younger children to develop adult-type PTB, which is considered more severe. Clinicians managing PTLD in adolescents should consider following the recommendations for the management of PTLD in adults.

**Table 7:** End-of-treatment assessment for post-TB lung disease in children and adolescents^*^

	Non-severe PTB^†^	Severe PTB
Clinical assessment and symptom/signs screening	X^‡^	X
Imaging (CXR)		X
Lung function test (spirometry)		X
6MWT		X
HRQoL		X

^*^ Source: Migliori G, et al.^46^

^†^ Applicable only to acid-fast bacilli smear-negative case; defined as PTB confined to one lobe with no cavities (<1 lobe), no signs of miliary TB and no complex pleural effusion, intrathoracic lymph node TB with no significant airway obstruction and no bilateral airway narrowing and peripheral lymph node TB.^227^

^‡^ Further investigations should be performed if there are any residual symptoms.

PTB = pulmonary TB; CXR = chest X-ray; 6MWT = 6-min walk test; HRQoL = health-related quality of life.

CXR: CXR should be considered at the end of treatment in children with more severe forms of disease, to assess residual abnormalities and to allow for future comparison if TB recurs or if residual symptoms persist. It is important to note that, in some cases, radiological improvement takes longer than 6 months to resolve.

Chest CT: CT is usually not widely available in resource-limited settings and is not routinely indicated. However, CT scans should be considered in specific cases where there are substantial chronic/recurrent respiratory symptoms and radiological abnormalities, to evaluate the extent of PTLD, or to exclude another underlying alternative diagnosis, including persistent TB disease.

Spirometry: Spirometry should be considered in all children with a history of severe PTB who are old enough to complete testing (typically 4 years and older).^46^ Spirometry should be conducted pre- and post-bronchodilation according to ATS/ERS guidelines^121^,^229^ using GLI 2012 reference ranges.^122^

#### Management of PTLD in children and adolescents

There are no data to guide the medical management of PTLD in children, but tools used in children with other chronic respiratory illnesses can be considered in paediatric PTLD.^46^ Medical treatment and long-term follow-up of children and adolescents who have PTLD should be guided by symptoms, the spectrum of respiratory disease and additional investigations. Bronchodilator treatment may be effective in children with COPD, if responsive to inhaled bronchodilators, but evidence is still limited. Children should be referred to a respiratory clinic if available, for assessment of possible bronchiectasis and management.^230^ The role of pulmonary rehabilitation and airway clearance techniques require further investigation. Its use should be guided by symptoms. Pulmonary rehabilitation should also include exercise, education, nutrition, self-management activities and psychosocial support.^46^,^231^

### TBM sequelae in children and adolescents

TBM disproportionally affects children under the age of 5 years and is considered the most deadly and devastating form of TB in children.^194^,^232^ A systematic review and meta-analysis found the mortality in 1,363 children with TBM to be almost 20%, with only one third of children surviving without neurological sequelae.^233^ Undiagnosed or untreated TBM is uniformly fatal. However, in one study of 184 children with TBM, mortality was only 3.8% on a shorter 6-month, four-drug TBM regimen recently included in WHO guidance for TBM treatment in children.^227^,^234^ Despite the initial high morbidity, the long-term outcomes of children who survive TBM are not well documented. Data on long-term neurocognitive, functional and behavioural impairment are lacking, especially for those children without accompanying physical disability.^194^ One reason for this lack of data is the absence of well-validated assessment tools, appropriately developed for and normed across different geographical and cultural settings that document findings across different ages and populations.^198^ There is a critical need for data on long-term outcomes and interventions to mitigate the consequences of TBM in children and their family.

#### Primary and secondary prevention of TBM sequelae

The most important interventions to reduce post TBM sequelae in children are primary prevention of TB using bacille Calmette-Guérin (BCG) vaccination, TB preventive therapy where indicated, and earlier diagnosis of TBM. Post-TBM sequelae are mostly due to a dysregulated host immune response.^235^,^236^ Therefore, host-directed therapies are the most promising strategy to improve overall survival and long-term clinical outcomes. More data are needed to inform the treatment of childhood TBM.^237^

#### Clinical presentation of neurological disability after TBM in children and adolescents

Advanced clinical stage of disease at presentation is associated with worse outcomes. Overt neurological sequelae are seen in approximately 50–65% of children presenting with TBM stages 2a/b and 3.^233^ Long-term outcomes in children with TBM include cerebral palsy, vision impairment, hearing loss, cognitive impairment, chronic seizure disorder, behavioural disturbance and developmental disability.^238^ Severe functional and neurocognitive disability is seen in an estimated 12–26% of cases of TBM, often compounded by co-infection with HIV, requiring long-term intensive physical care and support.^188^,^234^,^239^,^240^

#### Assessment of neurological disability after TBM in children and adolescents

All children should be assessed at the end of TB treatment for symptoms and receive a clinical examination, including general neurological and neurodevelopmental assessment (Table 8).^198^ Routine neuroimaging is generally not indicated as there is a poor correlation between imaging findings at the end of treatment and outcome.^241^ Standardised assessments of neurodevelopmental, neurocognitive, functional and neuro-behavioural outcomes are generally based on age, specific domains tested and type of test (performance based, self/caregiver rating). There are various instruments available that can be used at the end of treatment, throughout schooling and beyond. However, developmental assessment tools have not been formally adapted for use in LMICs, nor have locally determined norms been developed. Therefore, interpretation of results requires careful consideration of local context. Other, locally developed, screening tools are also available. Besides neurological assessment, children with severe disabling sequelae should be assessed in a multidisciplinary team for general clinical care requirements post TBM (Table 9). This includes the need for constipation, dental and pain management, as well as feeding and nutritional assessment. Some children will need alternative feeding options such as percutaneous gastrostomy with or without gastric fundoplication to relieve gastro-oesophageal reflux.

**Table 8:** End-of-treatment testing for neurocognitive and functional impairment in children and adolescents with tuberculous meningitis (adapted from 198)^*^

Outcome	Measure
Neurodevelopmental	Minimal: Ages and Stages Questionnaire^250^ (age range: 0–5 years) Optimal: Bailey Scale of Infant Development^251^ (age range: 1–42 months) Mullen Scale Early learning^252^ (age range: 0–68 months)
Neurocognitive	Wechsler Intelligence Scales for Children^253^ (age range: 6–16 years) Kauffman Assessment Battery for children. 2^nd^ ed.^254^ (age range: 3–8 years)
Functional	Minimal: Modified Rankin Scale^255^ (age range: 1 years–adult) WHO Disability Assessment Schedule 2.0^256^ (age range: 12 years–adult) Optimal: Vineland Adaptive Behaviour Scale^257^ (age range: birth to adult)
Neurobehavioural	Minimal: Strengths and Difficulties Questionnaire^258^ (age range: 4–17 years) Child, parent, teachers’ forms

^*^ Assessments should be commenced 6–9 months after diagnosis and repeated long-term throughout schooling (2 and 5 years minimum). Note: The developmental assessment tools cited have not been formally adapted for use in low- and middle-income countries, nor have locally determined norms been developed. Therefore, interpretation of results requires careful consideration of the local context. An additional number of in-country locally developed screening tools are also available.

**Table 9:** End-of-treatment clinical assessment in children with tuberculous meningitis

Specialist	Post treatment assessment
Neurologist and neurodevelopmental specialist	Clinical assessment and signs/symptoms screen with additional referral as needed
	Seizure/epilepsy management
	Medical spasticity management
	6-monthly shunt assessment (for VP shunts)
	Neurological cognitive and functional impairment assessment
	Neuroimaging only if clinically indicated (MRI if available)
Ophthalmologist/audiologist	Vision impairment, hearing loss
Physiotherapist/occupational therapist	Spasticity management/transport needs (wheelchair/adapted stroller (buggy) specific for individual’s needs)
Speech therapist/surgeon/gastrointestinal specialist	Feeding options including PEG with/without fundoplication; constipation management
Endocrinologist	Endocrine assessment in case of thalamic injury
Dental	Management of dental caries if indicated
Social worker	Educational assessment, support, and school placement for learners with special educational needs

VP = ventriculoperitoneal; MRI = magnetic resonance imaging; PEG = percutaneous endoscopic gastrostomy.

#### Care of children and adolescents after TBM

Dedicated follow-up and a multidisciplinary approach are required to manage the sequelae of TBM. Educational, psychosocial and socio-economic support for children, adolescents, and their families is essential.

#### Hydrocephalus

Hydrocephalus is seen in about 80% of children with TBM stage 2 and 3. Of these, approximately 20% have a non-communicating type that requires cerebrospinal fluid diversion, either endoscopic third ventriculostomy or ventriculoperitoneal shunting. Follow-up 6-monthly reviews of possible shunt dysfunction are required after ventriculoperitoneal shunting.

#### Spasticity and functionality

Physiotherapists or occupational therapists, preferably both, should be involved to assess and manage spasticity, promote functionality and assess the need for a wheelchair or adapted stroller (often referred to as a buggy).

#### Blindness

Approximately 15% of TBM survivors (all age groups) are either completely or partially blind.^190^ The main causes are 1) chronically raised intracranial pressure due to hydrocephalus and/or tuberculomas, 2) direct involvement of the optic chiasm or optic nerves, which may be transient if treated early, and 3) vasculitis related to occipital infarction, which is inevitably permanent. Regular evaluation of vision (visual acuity and fields) and general promotion of eye health is recommended.

#### Growth retardation

A proportion of children develop hypopituitarism due to TBM lesions affecting the hypothalamus or pituitary, which often only present years after recovery from TBM. Careful follow-up with endocrine assessment and early recognition of growth restriction, precocious puberty and obesity is important.

### Post-TB osteoarticular disease in children and adolescents

#### Presentation of post-TB osteoarticular disease in children and adolescents

Approximately 1–2% of all paediatric TB and 8% of EPTB in children manifests as osteoarticular disease. Despite this low incidence, the potential long-term consequences of the disease can be substantial.^242^

#### Management of post-TB osteoarticular disease in children and adolescents

Half of cases of osteoarticular disease are due to spinal TB, which can result in bone loss, deformity (kyphosis, kyphoscoliosis), secondary risk of neurological sequel-ae (e.g., paralysis) and disturbed overall growth potential.^190^,^241^,^243^,^244^ Even after successful treatment completion, spinal deformity and neurological sequel-ae can develop due to the growing skeleton. These children should therefore be followed annually at least until skeletal maturity (approximately 18 years of age) to check for potential progressive deformity, which could lead to late skeletal, neurological, cardiopulmonary and psychological complications. For other forms of osteoarticular TB, the conservation of overall functionality is the most important feature, including maintaining the range of motion, preventing contractures and assessing for joint destruction.^245^ This may require surgery, braces, physiotherapy or family-led rehabilitation. Management by a multidisciplinary team is needed (Table 10).

**Table 10:** Practical guidance to mitigate and assess post-TB morbidity in children with osteoarticular TB

Specialist	Task during/after TB treatment to prevent morbidity
Clinician	Review for contractures, joint destruction and recurrent TB
	Repeat X-ray 3-monthly (as clinically indicated) and at end of treatment to assess joint integrity
	Long-term follow-up for spinal TB for further collapse until age 18 years; may require post-TB spinal procedure in some cases
Physiotherapist	From diagnosis to maintain the range of motion and prevent contractures
Occupational therapist	To provide night splints to immobilise the joints in functional positions
Dietician	To improve nutritional status, including vitamin supplementation

### Post-TB quality of life and wellbeing in children and adolescents

A recent WHO-commissioned review highlighted the fact that TB has multiple negative impacts on adolescents, which persist well beyond their successful treatment outcomes; however, the data on this are limited.^246^ These effects include prolonged effects on mental health, experience of stigma, negative impact on esteem and feelings of isolation, the breakdown of peer, family and romantic relationships, interrupted and diminished educational, training and employment opportunities, impediments to privacy, and increased poverty and food insecurity. HRQoL instruments provide a holistic approach to quantifying illness-associated morbidity and the impact of health interventions. Data on the long-term impact of TB on HRQoL in children and adolescents are lacking. There is currently no TB-specific HRQoL measure for young children (0–5 years of age). Examples of generic, non-disease-specific HRQoL tools that can be used in young children are the EQ-5D-Y (7–14 years) and the Toddler and Infant dimensions of Health-Related Quality of Life (TANDI; 0–3 years).^243^,^244^ The EQ-5D-Y is a widely used, self-report measure for children aged 7 years and older.^247^ The TANDI was developed as a parental proxy experimental version based on the EQ-5D-Y and can be used in children 0–3 years old.^248^,^249^ Understanding and assessing caregivers’ quality of life, in addition to that of children with TB, is important, especially in the case of young children, who spend most of their time with their caregiver. Poor quality of life may affect a caregiver’s ability to provide sufficient care for the child.

## 6. FUTURE RESEARCH NEEDS

### Post-TB economic, social and psychological wellbeing

**Figure 9:**
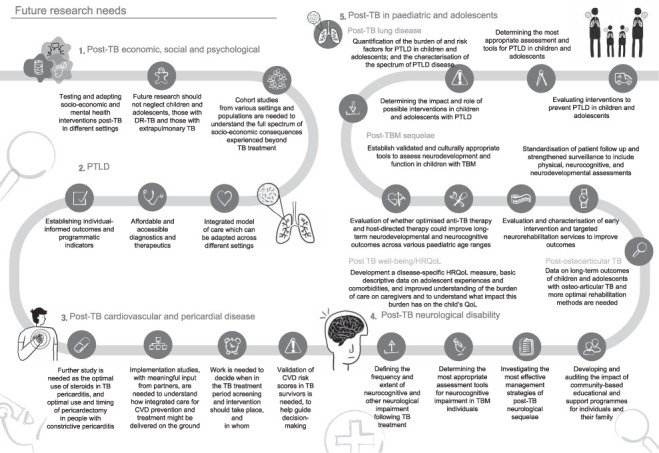
Future research needs. DR-TB = drug-resistant TB; PTLD = post-TB lung disease; TBM = TB meningitis; HRQoL = health-related QoL; QoL = quality of life; CVD = cardiovascular disease.

To ensure that life post-TB is considered as part of the process of treating TB, the following research priorities have been identified (see Figure 9):

- Testing and adapting socio-economic and mental health interventions post-TB in different settings will be an essential step to ensure the well-being of TB survivors and their reintegration into the economic and social spheres of life;
- While post-TB work has mainly focused on adults who have survived PTB, future research should not neglect children and adolescents, those with MDRTB and those with EPTB;
- Cohort studies from various settings and populations, including children, adolescents and those with drug resistance, are needed to understand the full spectrum of socio-economic consequences experienced beyond TB treatment.

### PTLD

Over the past decade, PTLD has taken a large step forward in terms of available research, with new publications helping us to understand the natural history and treatment of PTLD and the publication of clinical standards for the management of PTLD.^46^ However, evidence gaps remain. We suggest the following research priorities:

- Establishing person-centred informed outcomes and programmatic indicators, and understanding which outcomes are relevant across different settings;
- Affordable and accessible diagnostics, including non-sputum-based point of care tests for both diagnosis and monitoring of TB recurrence and alternative respiratory differential diagnoses. Data on the accuracy and performance of new diagnostic methods in this specific group of individuals will be essential;
- Affordable and accessible therapeutics (both pharmacological and non-pharmacological);
- Integrated model of care which can be adapted across different settings to manage risk of TB recurrence in PTLD, and assessment and management of other associated complications and conditions;
- Implementation of screening, diagnostic and treatment approaches in various programmatic settings.

### Post-TB cardiovascular and pericardial disease

There are several key research gaps around the primary prevention and management of CVD among TB survivors:

- Validation of CVD risk scores in TB survivors is needed to help guide decision-making around the use of pharmacological approaches to risk factors control and treatment targets required;
- For conditions such as hypertension and dyslipidaemia, further work is needed to decide when in the TB treatment period screening and intervention should take place, and in whom. Early interventions during TB treatment may be more effective, as they provide cover at a time when TB-related inflammation is most active and would allow serial TB treatment visits to be used to up-titrate doses and review compliance. However, this approach would increase the pill burden for individual receiving active TB treatment, risk interactions with TB medications, and may be challenging as an individuals’ clinical conditions change over the course of TB treatment. The risks and benefits of early vs. late introduction will need to be explored. Similarly, the potential role and best timing for considering statins and other host-directed therapies that may affect both TB and CVD risks needs further investigation.
- In the context of vertical TB programming, implementation studies, with meaningful input from individuals’ partners, are needed to understand how integrated care for CVD prevention and treatment might be delivered on the ground. While it may be possible to leverage the robust clinical pathways, staffing and data collection approaches of National TB Control Programmes to deliver CVD risk prevention for individuals receiving TB treatment, further work is required to understand whether this is a sustainable approach, how this might be supported by broader health services and how long-term care might be provided to individuals beyond the point of TB treatment completion.
- Several randomised controlled trials in TB pericarditis have been performed, but further study is needed as the optimal use of steroids in TB pericarditis remains uncertain. In addition, the optimal use and timing of pericardectomy in people with constrictive pericarditis requires further exploration.

### Post-TB neurological disability

There is clear evidence that neurological impairment post TB can occur as a direct result of infection due to the inflammatory response to TB, and as an adverse effect of treatment. However, there is a lack of research on the sequelae of neurological TB. Pressing research priorities include the following:

- Defining the frequency and extent of neurocognitive and other neurological impairment following TB treatment for neurological TB and other forms of TB;
- Determining the most appropriate assessment tools for neurocognitive impairment in individuals with TBM;
- Investigating the most effective management strategies of post-TB neurological sequelae;
- Developing and auditing the impact of community-based educational and support programmes for individuals and their family members.

### Post-TB health in children and adolescents

There are important research gaps across the spectrum of post-TB outcomes for children and adolescents and priorities include:

- Quantification of the burden of and risk factors for PTLD in children and adolescents;
- Characterisation of the spectrum of PTLD disease;
- Determining the most appropriate assessment and tools for PTLD in children and adolescents;
- Determining the impact and role of possible interventions in children and adolescents with PTLD;
- Evaluating interventions to prevent PTLD in children and adolescents.

Priorities for research on post-TBM sequelae in children and adolescents include:

- Establishing validated and culturally appropriate tools to assess neurodevelopment and function in children with TBM;
- Standardisation of follow-up and strengthened surveillance to include physical, neurocognitive and neurodevelopmental assessments;
- Evaluation of whether optimised anti-TB therapy and host-directed therapy could improve long-term neurodevelopmental and neurocognitive outcomes across various paediatric age ranges;
- Evaluation and characterisation of early intervention and targeted neurorehabilitation services to improve long-term outcomes.

Priorities for research on post-TB osteoarticular disease in children and adolescents include collecting data on long-term outcomes of children and adolescents with osteo-articular TB and more optimal rehabilitation methods are needed. Priorities for research on post-TB wellbeing in children and adolescents include:

- Development of a disease-specific HRQoL measure for children/adolescents with TB;
- Improved understanding of the burden of care on caregivers providing care for children with TB and other respiratory illnesses and the impact this burden has on the child’s quality of life;
- Basic descriptive (quantitative and qualitative) data on the experiences of adolescents before and after treatment completion, by sex, age, and disease profile, in the context of comorbidities, including HIV, and from diverse settings.

## CONCLUSIONS

This first clinical statement is limited by the lack of available research on both the natural history of post-TB mortality and treatments or interventions to improve the lives of TB survivors. Nevertheless, we offer this clinical statement as a resource for improving the care of those living with the long-term consequences of TB disease. We recognise that there is a global imbalance in the populations from which available data are sourced, but hope these gaps will be filled before the next version of this statement. We intend to update this document at regular intervals, in line with the vision to hold post-TB symposiums every 3–5 years, with the ultimate goal of developing robust evidence-based guidelines for sustainable post-TB health programmes.
